# Correction: Necrotrophism Is a Quorum-Sensing-Regulated Lifestyle in *Bacillus thuringiensis*

**DOI:** 10.1371/journal.ppat.1006049

**Published:** 2016-11-29

**Authors:** Thomas Dubois, Karoline Faegri, Sébastien Gélis-Jeanvoine, Stéphane Perchat, Christelle Lemy, Christophe Buisson, Christina Nielsen-LeRoux, Michel Gohar, Philippe Jacques, Nalini Ramarao, Leyla Slamti, Anne-Brit Kolstø, Didier Lereclus

Through the analysis of the microarrays of the *Bacillus thuringiensis* (*Bt*) *ΔkrsABC* mutant strain, the authors have come to understand that the experiments presented in the original article on the role of the kurstakin resulted from a mistake in the mutant strain *krsABC*. An analysis of the *krsABC* mutant strain revealed that the disruption of the *krsABC* by a *spc* gene, conferring resistance to spectinomycin, was introduced in the *Bacillus cereus* strain ATCC14579 instead of the *Bt* strain 407 Cry^-^. The *B*. *cereus* strain ATCC14579 is closely related to the *Bt* strain 407 Cry^-^ and is currently used in the authors’ laboratories for experiments focusing on *B*. *cereus*. A major difference between these two strains is the disruption of the *nprR* gene by a transposon in the *B*. *cereus* strain ATCC14579. As a result, this strain has naturally a NprR^-^ phenotype and should therefore be unable to survive in the insect cadavers like the *Bt* mutant strain 407 Δ*nprR* Δ*nprX* [1]. Moreover, it was previously shown that the *B*. *cereus* strain ATCC14579 did not produce biofilm in LB peptone medium [2] and did not swarm on EPS agar, a low-nutrient medium [3].

The error in the experiments described in Figure 5B might therefore be due to the lack of a functional NprR regulator in the *B*. *cereus* strain ATCC14579 instead of the disruption of the *krsABC* genes in the *Bt* strain 407 Cry^-^. In order to check this hypothesis, the authors attempted to disrupt the *krsABC* genes in the *Bt* strain 407 Cry^-^. Several unsuccessful experiments suggested that it was impossible to disrupt these three genes by using their methodology. Thus, the authors decided to disrupt the *krsC* gene coding for a major synthetase of the NPRS system. The deletion of this gene abolishes the production of kurstakin [4]. To investigate the role of the kurstakin during the late stage of infection, the authors monitored the growth of the wild-type and Δ*krsC Bt* 407 Cry^-^ strains in insect larvae (Figure 1). The results showed that the deletion of the *krsC* gene and the subsequent lack of kurstakin did not affect the survival of the *krsC* mutant strain for at least 3 days in the insect cadaver.

The conclusion that lipopeptide kurstakin is necessary for the survival of the wild type *Bt* strain 407 Cry^-^ in the insect cadavers is therefore incorrect.

The conclusions of this publication that remain valid are as follows:

1. During infection of an insect host, the quorum sensor NprR is active after death of the insect and allows *Bt* to survive in the cadavers as vegetative cells.
2. Transcriptomic analysis revealed that NprR regulates at least 41 genes, including many encoding degradative enzymes or proteins involved in the synthesis of a lipopeptide named kurstakin.
3. Constitutive expression of the *krs* operon involved in kurstakin production restored the survival of a *nprR* deficient mutant strain in the insect cadaver.
4. Necrotrophism is essential for the *Bt* infectious cycle, contributing to spore spreading.

To properly correct the original article, the authors have made substantial changes to the following sections: Author Summary, Results, Discussion, Materials and Methods, Figures, and References.

Additional changes include updating of the affiliations of the authors listed on the original article to acknowledge where the work for this correction was performed. Stéphane Perchat, Christelle Lemy, Christophe Buisson, Christina Nielsen-LeRoux, Michel Gohar, Nalini Ramarao, and Didier Lereclus are now affiliated with Micalis Institute, INRA, AgroParisTech, Université Paris-Saclay, 78350 Jouy-en-Josas, France. The present address of Thomas Dubois is: Unité Matériaux et Transformations, CNRS, INRA, Université de Lille, Villeneuve d’Ascq, France. His mail address is: Thomas.Dubois@lille.inra.fr.

Further changes include the addition of Sébastien Gélis-Jeanvoine and Leyla Slamti to the author list of this correction. Sébastien Gélis-Jeanvoine is listed as the third author and his affiliation is Micalis Institute, INRA, AgroParisTech, Université Paris-Saclay, 78350 Jouy-en-Josas, France. His mail address is: sebastien@gelis.ch. The contributions of this author are as follows: conceived and designed the experiments, performed the experiments and analyzed the data. SGJ declares no competing interests. SGJ received funding from the French “Ministère de l’Enseignement Supérieur et de la Recherche”.

Leyla Slamti is listed as the eleventh author and her affiliation is Micalis Institute, INRA, AgroParisTech, Université Paris-Saclay, 78350 Jouy-en-Josas. Her mail address is: Leyla.Slamti@jouy.inra.fr. The contributions of this author are as follows: conceived and designed the experiments, and analyzed the data. LS declares no competing interests. LS received funding from INRA.

All changes to the original article are detailed in a marked-up PDF included as S1 File. A clean Word document version of the revised article is included as S2 File. The renumbered Figures, including the corrected Fig 5, are included as S3 File.

## Supporting Information

S1 File Marked-up PDF detailing changes. (PDF)

S2 File Clean Word version of revised article. (DOC)

S3 File Zip file containing renumbered Figures. (ZIP)
