# Supplementary figures and images for: Necrotrophism Is a Quorum-Sensing-Regulated Lifestyle in Bacillus thuringiensis
Source: PLoS Pathog. 2012 Apr 12;8(4):e1002629. doi: 10.1371/journal.ppat.1002629 (PMC3325205; doi:10.1371/journal.ppat.1002629)

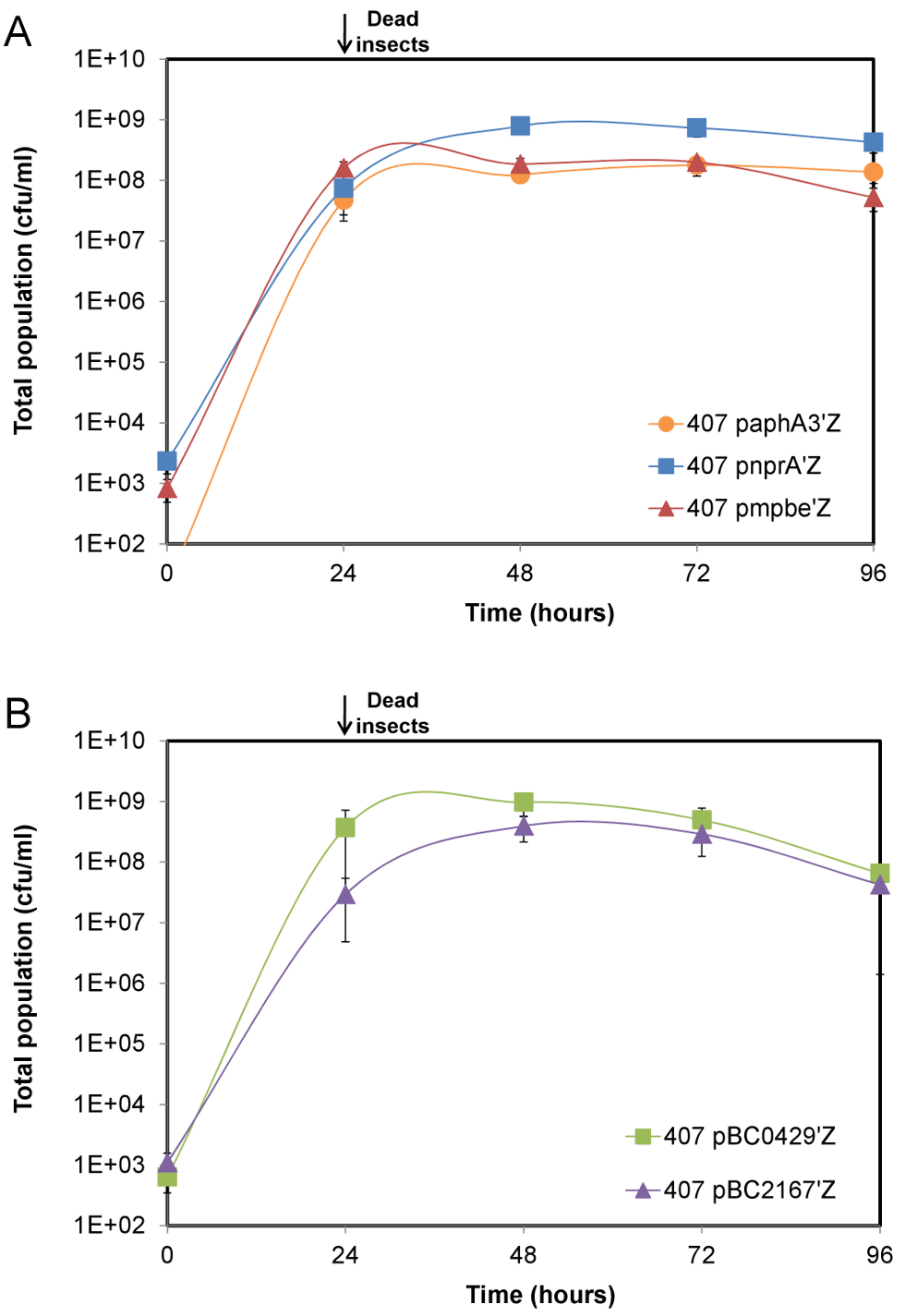

Supplement: Figure S1 — Growth kinetics of reporter strains. (A) Total population of the 407 paphA3′Z, 407 pnprA′Z and 407 pmpbE′Z strains in the insect larvae. (B) Total population of the 407 pBC0429′Z and 407 pBC2167′Z strains in the insect larvae. Data are averages of at least three independent experiments (error bars are SEM from mean values). (TIF) [file ppat.1002629.s001.tif]

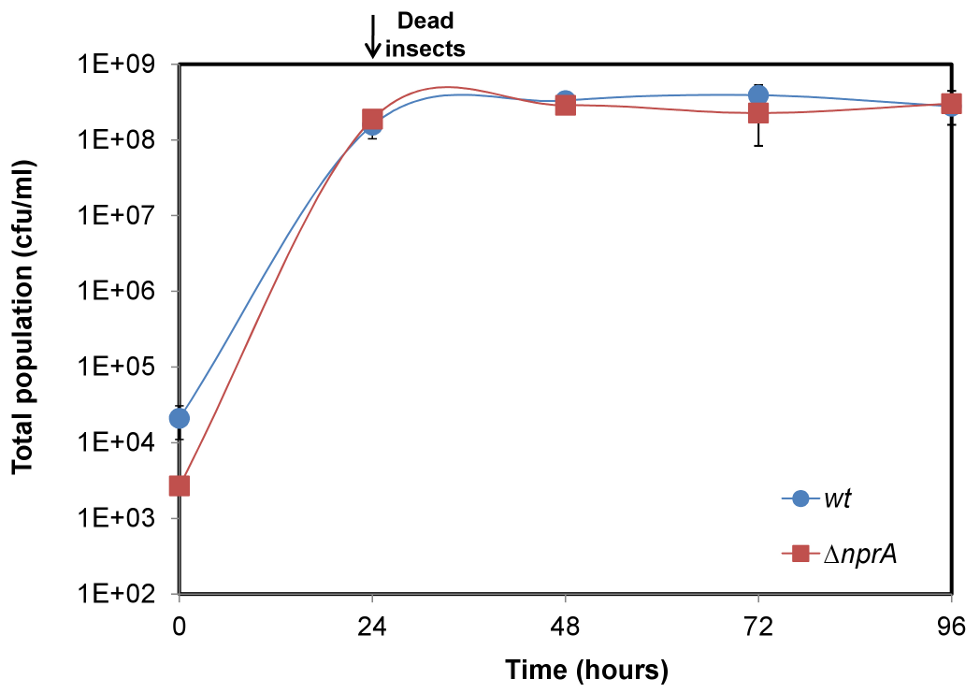

Supplement: Figure S2 — nprA is not required for the necrotrophic lifestyle of Bt . The total population of the ΔnprA strain was similar to that of the wt strain throughout the experiment indicating that nprA is not required for Bt to survive in the insect host. Data are averages of at least four independent experiments (error bars are SEM from mean values). (TIF) [file ppat.1002629.s002.tif]

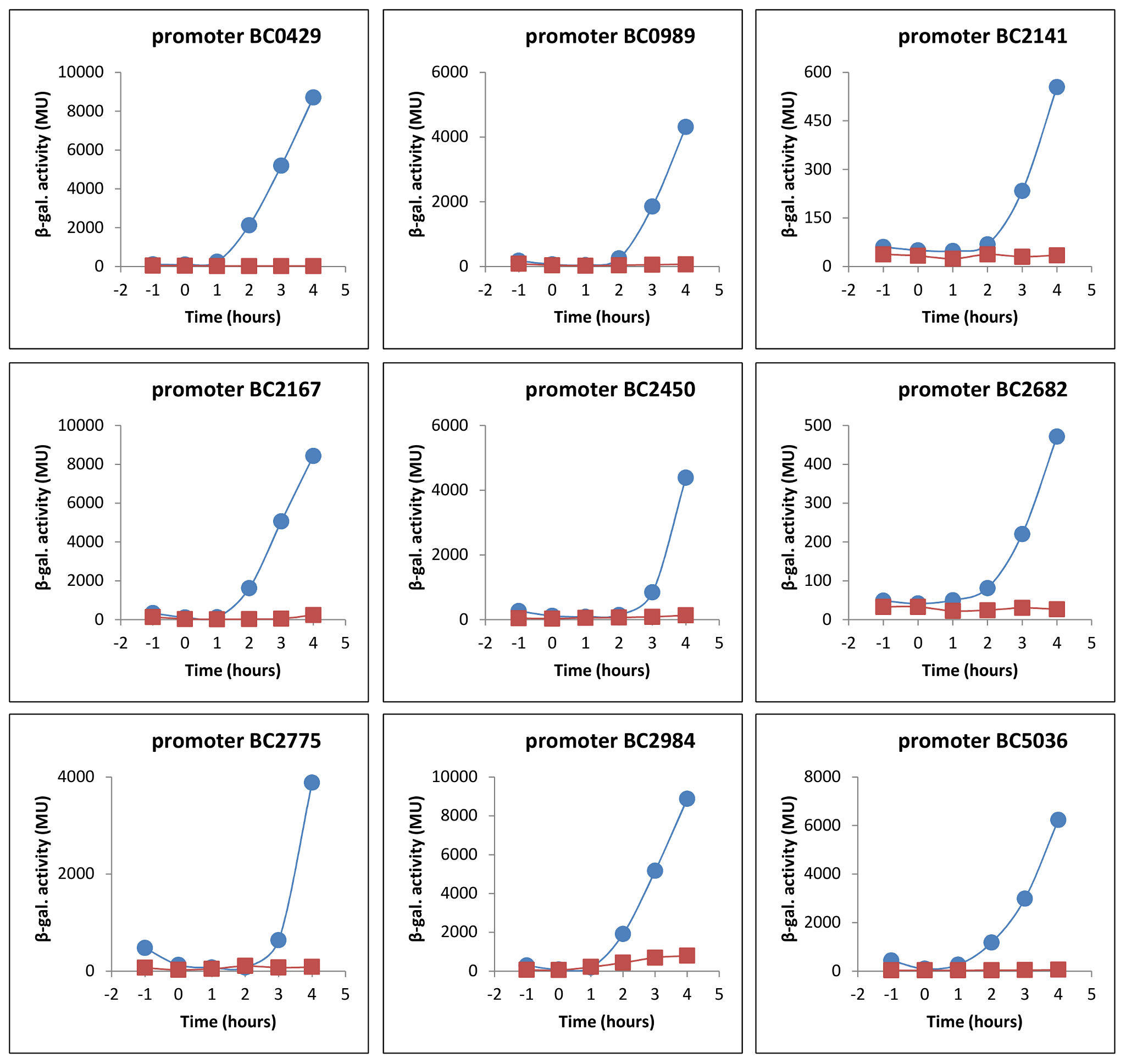

Supplement: Figure S3 — Differential expression of nine genes was confirmed with lacZ fusions. Fusions of the promoter region of the genes tested to a lacZ reporter on the plasmid pHT304-18Z were introduced into the wt (circles) and the ΔRX strains (squares), and expression was measured. Time on the x-axis is given relative to the transition to stationary phase (t0). β-Galactosidase activity in Miller units (MU) is plotted on the y-axis. Each assay was repeated at least twice independently and a representative graph is shown for each experiment. (TIF) [file ppat.1002629.s003.tif]

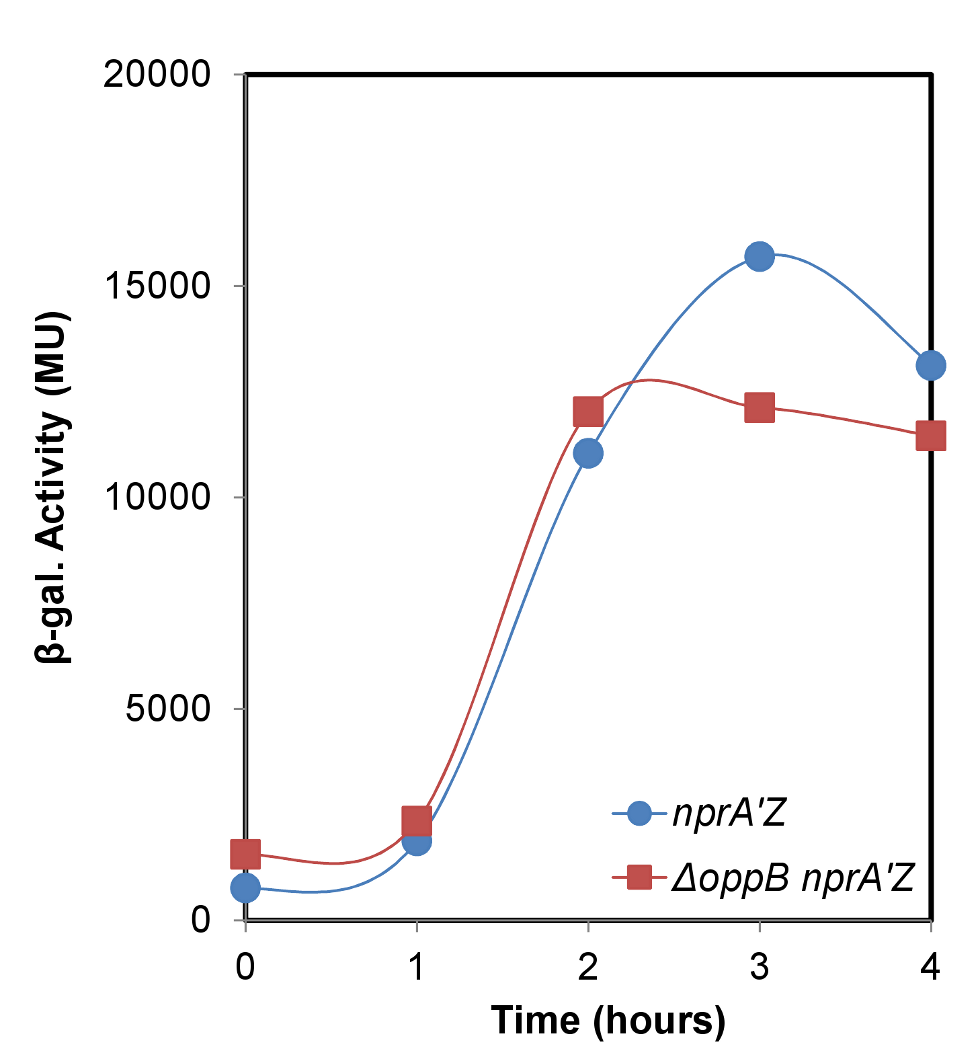

Supplement: Figure S4 — Expression of the P nprA - lacZ chromosomal fusions into the wt (circles) and the ΔoppB strains (squares). Time on the x-axis is given relative to the transition to stationary phase (t0). β-Galactosidase activity in Miller units (MU) is plotted on the y-axis. Assays were repeated at least three times independently and a representative graph is shown. (TIF) [file ppat.1002629.s004.tif]
