# Supplementary material for: Necrotrophism Is a Quorum-Sensing-Regulated Lifestyle in Bacillus thuringiensis
Source: PLoS Pathog. 2012 Apr 12;8(4):e1002629. doi: 10.1371/journal.ppat.1002629 (PMC3325205; doi:10.1371/journal.ppat.1002629)
Supplement: Table S1 — NprR is not involved in the pathogenicity of Bt . (DOC) [file ppat.1002629.s005.doc]

| **Strains** | **Force-feeding** | | **Injection** | |
| --- | --- | --- | --- | --- |
|  | **LD_50_** | **CI (95%)** | **LD_50_** | **CI (95%)** |
| wt | 7.0 x 10^6^ | 5.1 x 10^6^ – 9.0 x 10^6^ | 2.8 x 10^3^ | 2.3 x 10^3^ – 3.3 x 10^3^ |
| ∆RX | 8.2 x 10^6^ | 5.9 x 10^6^ – 10.6 x 10^6^ | 2.8 x 10^3^ | 2.2 x 10^3^ – 3.3 x 10^3^ |

CI : confidence intervals
