# Supplementary material for: Necrotrophism Is a Quorum-Sensing-Regulated Lifestyle in Bacillus thuringiensis
Source: PLoS Pathog. 2012 Apr 12;8(4):e1002629. doi: 10.1371/journal.ppat.1002629 (PMC3325205; doi:10.1371/journal.ppat.1002629)
Supplement: Table S2 — The ΔRX strain is unable to sporulate in the insect host. These experiments were done at 30°C. The percentages were calculated as 100× the ratio between heat-resistant spores ml−1 and viable cells ml−1. For both strains, the OD600 at t0 in HCT medium was 2.2±0.1 and that in LB was 2.6±0.1. n is the number of independent sporulation efficiency measurements. Results are given as mean ± SEM. (DOC) [file ppat.1002629.s006.doc]

| **strains** | **Medium** | **Time (hours)** | **Viable counts ml−1** | **Heat-resistant spores ml−1** | **% spores** | **n** |
| --- | --- | --- | --- | --- | --- | --- |
| wt | HCT | 48 | 4.49E+08 ± 3.82E+07 | 3.80E+08 ± 3.89E+07 | 85 ± 2 | 3 |
|  | LB | 72 | 2.69E+08 ± 1.81E+07 | 1.58E+08 ± 1.13E+07 | 59 ± 1 | 7 |
|  | Insects | 0 | 2.09E+04 ± 9.85E+03 | 0.00E+00 ± 0.00E+00 | 00 ± 0 | 7 |
|  |  | 24 | 1.56E+08 ± 5.33E+07 | 5.57E+02 ± 1.68E+02 | 00 ± 0 | 7 |
|  |  | 48 | 3.31E+08 ± 5.13E+07 | 1.05E+08 ± 3.66E+07 | 32 ± 12 | 7 |
|  |  | 72 | 3.91E+08 ± 1.43E+08 | 7.24E+07 ± 2.36E+07 | 19 ± 8 | 6 |
|  |  | 96 | 2.78E+08 ± 5.22E+07 | 5.53E+07 ± 1.89E+07 | 20 ± 6 | 7 |
| ΔRX | HCT | 48 | 4.56E+08 ± 1.88E+07 | 3.46E+08 ± 7.78E+06 | 76 ± 2 | 5 |
|  | LB | 72 | 1.77E+08 ± 2.42E+07 | 8.30E+07 ± 1.43E+07 | 47 ± 2 | 7 |
|  | Insects | 0 | 1.47E+02 ± 4.99E+01 | 0.00E+00 ± 0.00E+00 | 00 ± 0 | 5 |
|  |  | 24 | 1.17E+08 ± 5.60E+07 | 1.00E+00 ± 1.00E+00 | 00 ± 0 | 5 |
|  |  | 48 | 5.83E+05 ± 3.96E+05 | 2.00E+02 ± 2.00E+02 | 00 ± 0 | 5 |
|  |  | 72 | 9.54E+02 ± 9.24E+02 | 0.00E+00 ± 0.00E+00 | 00 ± 0 | 5 |
|  |  | 96 | 2.42E+01 ± 2.40E+01 | 0.00E+00 ± 0.00E+00 | 00 ± 0 | 5 |
