# Supplementary material for: Necrotrophism Is a Quorum-Sensing-Regulated Lifestyle in Bacillus thuringiensis
Source: PLoS Pathog. 2012 Apr 12;8(4):e1002629. doi: 10.1371/journal.ppat.1002629 (PMC3325205; doi:10.1371/journal.ppat.1002629)
Supplement: Table S3 — NprR is a pleiotropic regulator. The NprR-regulated genes can be classified into four functional groups encoding: stress resistance proteins (in purple); an oligopeptide permease (in green); a NRPS system (in orange); and food supply proteins (in blue). This table includes genes more than four times differentially regulated in ΔRX strain relative to the wt strain at t3 in LB medium, as confirmed by qRT-PCR (expression ratios). Locus tags for the Bc ATCC 14579 microarray probes are listed. (DOC) [file ppat.1002629.s007.doc]

| **Locus tag** | **Name** | **predicted localisation** | **expression ratios** |
| --- | --- | --- | --- |
| BC1061 | hypothetical protein | cytoplasmic | 0,034 |
| BC1062 | 3-oxoacyl-[acyl-carrier protein] reductase | cytoplasmic | 0,024 |
| BC1063 | transporter. Drug/Metabolite Exporter family | membrane | 0,177 |
| BC2066 | macrolide glycosyl transferase | cytoplasmic | 0,008 |
| BC2622 | macrolide glycosyltransferase | cytoplasmic | 0,130 |
| BC2617 | cysteine dioxygenase | cytoplasmic | 0,023 |
| BC2140 | N-hydroxyarylamine O-acetyltransferase | cytoplasmic | 0,008 |
| BC2613 | cytochrome P450 | cytoplasmic | 0,056 |
| BA3286 | peroxydase | extracellular | 0,029 |
| BC1180 | oligopeptide ABC transporter permease protein OppB | membrane | 11,472 |
| BC1181 | oligopeptide ABC transporter.permease protein OppC | membrane | 12,996 |
| BC1182 | oligopeptide ABC transporter ATP-binding protein OppD | membrane | 15,207 |
| BC1183 | oligopeptide ABC transporter ATP-binding protein OppF | membrane | 11,917 |
| BC2450 | macrolide-efflux protein | membrane | 0,012 |
| BC2451 | peptide synthetase | cytoplasmic | 0,008 |
| BC2452 | peptide synthetase | cytoplasmic | 0,012 |
| BC2453 | peptide synthetase | cytoplasmic | 0,010 |
| BC2454 | peptide synthetase | cytoplasmic | 0,014 |
| BC2455 | peptide synthetase | cytoplasmic | 0,018 |
| BC2456 | peptide synthetase | cytoplasmic | 0,017 |
| BC0602 | neutral protease NprA | extracellular | 0,006 |
| BC2167 | neutral protease | extracellular | 0,006 |
| BC2984 | immune inhibitor A precursor InhA3 | extracellular | 0,103 |
| BC5036 | neutral protease | extracellular | 0,005 |
| Bant_01003380 | esterase/Lipase | cytoplasmic | 0,197 |
| BC2141 | lipase | extracellular | 0,045 |
| BC2743 | carboxylesterase | cytoplasmic | 0,030 |
| BC0429 | endochitinase ChiCW | extracellular | 0,011 |
| BC2682 | chitosanase | extracellular | 0,004 |
| BC3725 | exochitinase ChiCH | extracellular | 0,031 |
| BC2827 | chitin-binding protein | extracellular | 0,059 |
| BC3526 | collagen adhesion protein | cell wall | 0,064 |
| BC1697 | asparagine synthetase. glutamine-hydrolyzing | cytoplasmic | 0,029 |
| BC1848 | prophage helix-turn-helix protein | cytoplasmic | 0,123 |
| BC1997 | intracellular serine protease | cytoplasmic | 0,117 |
| BC0989 | hypothetical protein | extracellular | 0,001 |
| BC1241 | hypothetical protein | cytoplasmic | 0,118 |
| BC1243 | hypothetical protein | membrane | 0,134 |
| BC1244 | hypothetical protein | membrane | 0,226 |
| BC2775 | hypothetical protein | extracellular | 0,001 |
| BC5360 | hypothetical protein | extracellular | 0,046 |
