# Supplementary material for: Necrotrophism Is a Quorum-Sensing-Regulated Lifestyle in Bacillus thuringiensis
Source: PLoS Pathog. 2012 Apr 12;8(4):e1002629. doi: 10.1371/journal.ppat.1002629 (PMC3325205; doi:10.1371/journal.ppat.1002629)
Supplement: Table S4 — Primers used for construction of recombinant strains and transcriptional fusions. Restriction sites are underlined. (DOC) [file ppat.1002629.s008.doc]

| **Primer name** | **Primer sequences** | **Restriction site** |
| --- | --- | --- |
| **NRPS1** | CCCAAGCTTCTATTATCCTGCACAGCAAACAC | *Hind*III |
| **NRPS2** | CGGGGTACCCCTTTATCAGAATGAACGACTTCT | *Kpn*I |
| **NRPS3** | GCTCTAGAGAGCTGTTTACAGATTGGGGAC | *Xba*I |
| **NRPS4** | CGCGGATCCGCTTGTATGAAATTAAAGTTCGTG | *Bam*HI |
| **NRPS C1** | CGCGGATCCGCAAAGCGTGGGTTAGTAACTA | *Bam*HI |
| **NRPS C2** | AACTGCAGATAGATGGCTTTAGCCATAAGGAT | *Pst*I |
| **NRPS C3** | CCGCGCATGCACCTTATACGCAAAAGCGTTTA | *Sph*I |
| **NRPS C4** | CCCAAGCTTAGATACAAGCATCAGCCCACT | *Hind*III |
| **pApha3-1** | GCATGCCTGCAGGTGATAAACC | *Pst*I |
| **pApha3-2** | GCTCTAGACAATTCCGGTGATATTCTCATTTTACCC | *Xba*I |
| **Kana Cfwd** | AACTGCAGCGAACCATTTGAGGTGATAG | *Pst*I |
| **Kana Crev** | CCGCGCATGCTAAATCTAGGTACTAAAACAATTCATCC | *Sph*I |
| **BC0429F** | CGCGGATCCAGTGTGAATTTTTGAGACCTCATAGC | *Bam*HI |
| **BC0429R** | CCCAAGCTTCCATTTCTGGTGGAATCATTCCCGC | *Hin*dIII |
| **BC0989F** | AACTGCAGTTAGTGCAAATATGTTATTCGGATC | *Pst*I |
| **BC0989R** | GCTCTAGATGCAATACCTGTTGCTAACGCTCC | *Xba*I |
| **BC2141F** | AACTGCAGAATTTTCTTCTGCTCACCGATAG | *Pst*I |
| **BC2141R** | GCTCTAGACCATTCTCCAGGTGTGAATACTTCTG | *Xba*I |
| **BC2167F** | AACTGCAGCACATAATTCTGTGAAAATACATTCG | *Pst*I |
| **BC2167R** | GCTCTAGAAATCGTCCCCATTACTAACGGC | *Xba*I |
| **BC2450F** | AACTGCAGCTTTTTGTTTAGTAGGATTTATAGTCTCT | *Pst*I |
| **BC2450R** | GCTCTAGACCAAGCATAAGCAATGAATAGG | *Xba*I |
| **BC2682F** | AACTGCAGGAATTGCCTTAATTTCATTTAG | *Pst*I |
| **BC2682R** | GCTCTAGAATTTTGTATTGAATTGTCCGTTAC | *Xba*I |
| **BC2775F** | AACTGCAGTTCACCTGATGGGTATTCTGAATTAG | *Pst*I |
| **BC2775R** | GCTCTAGAGCACTTACCGCACCTAGTCCTCC | *Xba*I |
| **BC2984F** | CGGGATCCAGCATGCGCTGCGTGACCTCC | *Bam*HI |
| **BC2984R** | CCCAAGCTTGTAACAGAGAATCACGCTATA | *Hin*dIII |
| **BC5036F** | GCTCTAGATAACAGTTAACGCTAATGCTGATG | *Xba*I |
| **BC5036R** | AACTGCAGCATAATTTAGGCGATGAAACGC | *Pst*I |
