# Supplementary material for: Necrotrophism Is a Quorum-Sensing-Regulated Lifestyle in Bacillus thuringiensis
Source: PLoS Pathog. 2012 Apr 12;8(4):e1002629. doi: 10.1371/journal.ppat.1002629 (PMC3325205; doi:10.1371/journal.ppat.1002629)
Supplement: Table S5 — Plasmids used in this study. (DOC) [file ppat.1002629.s009.doc]

| **Plasmid name** | **Plasmid features** |
| --- | --- |
| pRN5101Ω*krsABC*::*spc* | 5' and 3' regions of the NRPS locus (BC2451 to BC2456) were amplified using primers NRPS1/NRPS2 and NRPS3/NRPS4, respectively, and 407 chromosomal DNA as template. The 5' end was purified as a *Hin*dIII-*Kpn*I fragment and the 3' end as a *XbaI*–*Bam*HI fragment. A 1.1 kb *Xba*I-*Kpn*I fragment containing the spectinomycin resistance gene was purified from pUC18Ω*spc* and inserted with the 5' and 3' parts of the NRPS locus between the *Hin*dIII and *Bam*HI sites of pRN5101. |
| pRN5101ΩP*_krsABC_*::*aphA3* | 5' and 3' regions of the NRPS promoter region were amplified using primers NRPS C1/NRPS C2 and NRPS C3/NRPS C4, respectively, and 407 chromosomal DNA as template. The 5' end was purified as a *Bam*HI-*Pst*I fragment and the 3' end as a *SphI*–*Hin*dIII fragment. A 1.3 kb fragment containing the kanamycine resistance gene with *aphA3* promoter but without it final stern-loop structure was amplified by PCR using primers Kana Cfwd / Kana Crev from pDG783 (Guérout-Fleury *et* *al.*, 1995), purified as a *Pst*I-*Sph*I fragment and inserted with the 5' and 3' parts of the NRPS promoter region between the *Hin*dIII and *Bam*HI sites of pRN5101. |
| pRN5101Ω*nprA*::*lacZ* | This plasmid was previously described (Perchat *et al*., 2011). |
| pHT304-RX | This plasmid was previously described (Perchat *et al*., 2011). |
| paphA3*’*Z | The promoter region of *apha3* gene was amplified using primers pApha3-1/ pApha3-2 with pDG783 as template and inserted between the *Pst*I and *Xba*I sites of pHT304-18Z. This results in the creation of a transcriptional fusion between *apha3* promoter and *lacZ* gene. |
| pnprA’Z | This plasmid was previously described as pA1’Z (Perchat *et al*., 2011). |
| pmpbE’Z | This plasmid was previously described as pHT304mpbE’Z (Hajaij-Ellouze *et al*., 2006). |
| pBC0429’*Z* | The promoter region of BC0429 gene was amplified using primers BC0429F/ BC0429R with 407 chromosomal DNA as template and inserted between the *Hind*III and *Bam*HI sites of pHT304-18Z. This results in the creation of a transcriptional fusion between BC0429 promoter and *lacZ* gene. |
| pBC0989’*Z* | The promoter region of BC0989 gene was amplified using primers BC0989F/ BC0989R with 407 chromosomal DNA as template and inserted between the *Pst*I and *Xba*I sites of pHT304-18Z. This results in the creation of a transcriptional fusion between BC0989 promoter and *lacZ* gene. |
| pBC2141’*Z* | The promoter region of BC2141 gene was amplified using primers BC2141F/ BC2141R with 407 chromosomal DNA as template and inserted between the *Pst*I and *Xba*I sites of pHT304-18Z. This results in the creation of a transcriptional fusion between BC2141 promoter and *lacZ* gene. |
| pBC2167’*Z* | The promoter region of BC2167 gene was amplified using primers BC2167F/ BC2167R with 407 chromosomal DNA as template and inserted between the *Pst*I and *Xba*I sites of pHT304-18Z. This results in the creation of a transcriptional fusion between BC2167 promoter and *lacZ* gene. |
| pBC2450’*Z* | The promoter region of BC2450 gene was amplified using primers BC2450F/ BC2450R with 407 chromosomal DNA as template and inserted between the *Pst*I and *Xba*I sites of pHT304-18Z. This results in the creation of a transcriptional fusion between BC2450 promoter and *lacZ* gene. |
| pBC2682’Z | The promoter region of BC2682 gene was amplified using primers BC2682F/ BC2682R with 407 chromosomal DNA as template and inserted between the *Pst*I and *Xba*I sites of pHT304-18Z. This results in the creation of a transcriptional fusion between BC2682 promoter and *lacZ* gene. |
| pBC2775’Z | The promoter region of BC2775 gene was amplified using primers BC2775F/ BC2775R with 407 chromosomal DNA as template and inserted between the *Pst*I and *Xba*I sites of pHT304-18Z. This results in the creation of a transcriptional fusion between BC2775 promoter and *lacZ* gene. |
| pBC2984’Z | The promoter region of BC2984 gene was amplified using primers BC2984F/ BC2984R with 407 chromosomal DNA as template and inserted between the *Hind*III and *Bam*HI sites of pHT304-18Z. This results in the creation of a transcriptional fusion between BC2984 promoter and *lacZ* gene. |
| pBC5036’Z | The promoter region of BC5036 gene was amplified using primers BC5036F/ BC5036R with 407 chromosomal DNA as template and inserted between the *Pst*I and *Xba*I sites of pHT304-18Z. This results in the creation of a transcriptional fusion between BC5036 promoter and *lacZ* gene. |
