# Supplementary material for: Correction: Necrotrophism Is a Quorum-Sensing-Regulated Lifestyle in Bacillus thuringiensis
Source: PLoS Pathog. 2016 Nov 29;12(11):e1006049. doi: 10.1371/journal.ppat.1006049 (PMC5127582; doi:10.1371/journal.ppat.1006049)
Supplement: S2 File — (DOC) [file ppat.1006049.s002.doc]

**Necrotrophism is a quorum-sensing-regulated lifestyle in *Bacillus thuringiensis***

Thomas Dubois^1¶^, Karoline Faegri^2¶^, Sébastien Gélis-Jeanvoine^1^, Stéphane Perchat^1^, Christelle Lemy^1^, Christophe Buisson^1^, Christina Nielsen-LeRoux^1^, Michel Gohar^1^, Philippe Jacques^3^, Nalini Ramarao^1^, Leyla Slamti^1^, Anne-Brit Kolstø^2§^ and Didier Lereclus^1§^

^1^ Micalis Institute, INRA, AgroParisTech, Université Paris-Saclay, 78350 Jouy-en-Josas, France..

^2^School of Pharmacy, Laboratory for Microbial Dynamics, University of Oslo, N-0316 Oslo, Norway

^3^ProBioGEM, UPRES EA 1026, Université Lille Nord de France, USTL, F-59655 Villeneuve d'Ascq, France.

Key words: Biosurfactant, cell communication, regulation, saprophytism, sporulation

¶These authors contributed equally to this work

§To whom correspondence may be addressed. E. mail: Didier.Lereclus@jouy.inra.fr or a.b.kolsto@farmasi.uio.no

**Abstract**

How pathogenic bacteria infect and kill their host is currently widely investigated. In comparison, the fate of pathogens after the death of their host receives less attention. We studied *Bacillus thuringiensis* (*Bt*) infection of an insect host, and show that NprR, a quorum sensor, is active after death of the insect and allows *Bt* to survive in the cadavers as vegetative cells: a *nprR* deficient mutant does not develop necrotrophically and does not sporulate efficiently in the cadaver.. Transcriptomic analysis revealed that NprR regulates at least 41 genes, including many encoding degradative enzymes or proteins involved in the synthesis of a nonribosomal peptide named kurstakin. These degradative enzymes are essential *in vitro* to degrade several substrates and are specifically expressed after host death suggesting that *Bt* has an active necrotrophic lifestyle in the cadaver. Kurstakin is not essential for *Bt* survival during necrotrophic development. However, constitutive expression of the *krs* operon involved in kurstakin production restored the survival of a *npr* deficient mutant strain in the insect cadaver. We report that necrotrophism is a highly regulated mechanism essential for the *Bt* infectious cycle, contributing to spore spreading.

**Author summary**

*Bacillus thuringiensis* (*Bt*) is a well known entomopathogenic bacterium successfully used as a biopesticide for fifty years. The insecticidal properties of *Bt* are mainly due to specific toxins forming a crystal inclusion associated with the spore. After ingestion by susceptible insect larvae, toxins could induce favorable conditions for spore germination. The bacteria multiply in the insect and coordinate their behavior using signaling molecules involved in quorum sensing. The activation of the quorum sensor PlcR leads to the production of virulence factors allowing the bacteria to kill the insect host. Here we show that, in the cadaver, *Bt* shifts from a virulent to a necrotrophic lifestyle during which a second quorum sensor (NprR) becomes functional and is essential for the survival of *Bt* after insect death. NprR activates genes encoding degradative enzymes (proteases, lipases and chitinases) and a lipopeptide (kurstakin). This suggests that NprR allows the bacteria to survive and eventually to sporulate in the host cadaver, thus improving their ability to disseminate in the environment. Altogether these results show that the pathogenic and necrotrophic lifestyles of *Bt* are tightly controlled by two quorum-sensing systems acting sequentially during the infection process.

**Introduction**

Saprophytism, probably one of the most common lifestyle for micro-organisms, involves living in dead or decaying organic matter. For most pathogens, saprophytism is limited to necrotrophism (the use of the host cadaver). This step of the infection process is essential for the proliferation and horizontal transmission of these microorganisms (transfer of infection within a single generation) [1]. However, there have been very few studies addressing this major issue. The transition from a pathogenic to a necrotrophic lifestyle implies substantial metabolic changes for microorganisms [2]. The death of the host is a critical event which compels the micro-organisms to cope with a new series of challenges: competition with the commensal organisms and opportunistic incomers, stress, and nutrient deficiencies. Therefore, necrotrophism is likely to be highly regulated.

The insect pathogen *Bacillus thuringiensis* (*Bt*) is a suitable model for studying the time course of the infection process, including necrotrophism in the insect cadaver. *Bt* is an ubiquitous spore-forming bacterium belonging to the *Bacillus cereus* (*Bc*) group [3]. Its spores are found in a large variety of environments, such as soils, dead and living insects and plant phylloplane [4]. However, *Bt* probably does not grow in soil and reports of natural epizootic episodes are very rare [5,6]. Unlike soil bacteria, such as *Streptomyces spp* and *B. subtilis*, *Bc* group genomes contain a large number of genes involved in nitrogen metabolism [3]. It is therefore likely that *Bt* multiplies in the host cadaver [1,6].

*Bt* carries plasmids encoding specific insecticidal toxins responsible for their insecticidal properties [7]. *Bt* spores and toxins are ingested by larvae, and the toxins bind to specific receptors on the midgut epithelial cells, inducing cell lysis and creating favorable conditions for the development of the bacteria [8]. The vegetative bacteria multiply in the insect hemocoel and cause septicemia [1,9]. *Bt* also harbors genes encoding exported virulence factors including enterotoxins, hemolysins, phospholipases and proteases [10]. The transcription of most of these virulence genes in bacteria growing in a rich medium is activated at the onset of stationary phase by the quorum-sensing system PlcR-PapR [11,12]. PlcR-regulated factors account for about 80% of the extracellular proteome of *Bt* during early stationary phase in rich medium [13]. In sharp contrast, the expression of the PlcR-regulated genes is repressed when the bacteria enter sporulation [14] and the stationary phase secretome of *Bt* and *B. anthracis* (*Ba*) growing in a sporulation medium is mainly composed of the metalloprotease NprA [15,16]. NprA (also designated NprB and Npr599 in *Ba*) cleaves tissue components such as fibronectin, laminin and collagen, thus displaying characteristics of pathogenic factors [17]. Transcription of *nprA* is activated during the late stationary phase by the regulator NprR [16]. NprR is a quorum sensor activated by its cognate signaling peptide, NprX. NprR-NprX functions as a typical Gram-positive quorum-sensing system: the pro-signaling peptide NprX is exported from the cell, and after being processed to its active form is reimported, and binds to NprR allowing the recognition of its DNA target and the activation of *nprA* transcription [16].

The first stages of *Bt* infection are relatively well documented, but the fate of the bacteria after death of the host remains unclear. Here, we report evidence that the necrotrophic lifestyle of *Bt* is a specific and highly regulated process. The quorum-sensing system NprR-NprX controls at least 41 genes some of which are required for *Bt* to survive in the insect cadaver and to complete its development *in vivo* ending with the production of spores.

##### Results

**NprR is activated after the host death.**

We tested whether NprR, the activator of *nprA* transcription [16], is involved in the pathogenicity of *Bt*. The LD_50s_ of the *Bt* 407 Cry^-^ (wt) strain and of the *nprR*-deficient (∆RX) strain in the insect model *Galleria mellonella* were measured in two ways: by feeding larvae with spores mixed with the insecticidal toxin Cry1C and by injection of vegetative bacteria into the insect hemocoel (Table S1). The LD_50s_ of two strains did not differ significantly in either of the two conditions indicating that NprR was not required for pathogenicity. Consistently, an *nprA*-deficient strain was similarly found not to be affected in pathogenicity (not shown).

We investigated the involvement of NprR in the infection process by comparing, *in vivo*, the expression kinetics of *nprA* with that of the protease gene *mpbE*, reflecting the transcriptional activities of NprR and PlcR, respectively [16,18]. The reporter strains grew similarly in insect larvae and a constitutively expressed P*_aphA3_-lacZ* fusion was used as the reference standard (Figure 1A and Figure S1A). Transcription of *mpbE* increased between 0 h and 24 h after injection and gradually decreased thereafter. In contrast, *nprA* transcription was low between 0 h and 24 h, increased between 24 h and 48 h and then decreased sharply (Figure 1B). Thus, NprR is active later in the infection process than PlcR, and after the death of the host.

**NprR allows *Bt* to survive in insect cadavers by a process independent of sporulation.**

To investigate the role of NprR during the late stage of infection, we compared the growth of the wt and ∆RX strains in insect larvae (Figure 2A). The total population of the two strains increased between 0 h and 24 h to reach about 1 x 10^8^ cfu/mL. From 24 h to 96 h, the population of the wt strain remained stable, whereas the population of the ∆RX strain decreased sharply: 96 h post infection, the total population of the ∆RX strain was 6-log lower than that of the wt strain. Complementation of the ∆RX strain by pHT304-RX restored the wt phenotype. These findings indicate that NprR substantially improves the survival of *Bt* in insect cadavers.

In sporulating microorganisms, sporulation is generally regarded as the key process ensuring survival in unfavorable conditions. We therefore investigated i) whether NprR was involved in the sporulation process of *Bt* in the insect cadaver, and ii) whether sporulation is responsible for the survival of the bacteria in the insect cadaver. We compared the sporulation efficiencies of the wt and ∆RX strains in both LB and sporulation-specific medium (HCT) (Table S2). In HCT, the sporulation efficiencies of the two strains were similar. However, in LB medium, the total number of viable spores of the ∆RX strain was half that for the wt strain (8.30 x 10^7^ *vs* 1.58 x 10^8^), suggesting that NprR is involved in the sporulation of *Bt* in rich medium. Next, we monitored the counts of wt and ∆RX strain spores in insect larvae over 96 h (Figure 2B and Table S2). For the wt strain, heat-resistant spores were detected 24 h after injection and their number increased until 48 h. From 48 h to 96 h, the number of spores remained stable and represented one third of the total bacterial population. The large number of non sporulated bacteria 96 h after the death of the insect suggests that sporulation was not the main mechanism allowing *Bt* to survive. For the ∆RX strain, less than one percent of the bacterial population was heat-resistant spores throughout the infection process. The decrease in the number of heat-resistant spores from 48 h to 72 h is likely due to the germination of the spores. We suspected that the low number of spores is not a cause but a consequence of the inability of the ∆RX strain to survive in the insect cadaver. To test this idea, we tested the survival of a *sigK*-deficient strain (Figure 2C): SigK is a sigma factor involved in the transcription of late sporulation genes in the mother cell, and *sigK*-deficient strains are not able to form viable spores [19,20]. The total population of the *sigK* strain in the insect cadaver was similar to the total population of the wt strain, indicating that NprR ensures the survival of *Bt* by a process independent of sporulation.

## NprR is a pleiotropic regulator involved in the necrotrophic development of *Bt* in the insect cadaver.

The only gene described as being controlled by NprR was *nprA*. Therefore, we monitored the survival of a ∆*nprA* strain in infected larvae (Figure S2). The survival of the wt and ∆*nprA* strains was similar throughout the experiment, suggesting that other NprR-regulated genes are involved in bacterial survival. Microarray analysis was used to identify other NprR-regulated genes. Gene expression ratios between the wt and the ∆RX strains were determined 3 h after the onset of stationary phase (t3), when *nprA* transcription increases sharply [16]. For 107 genes, this expression ratio was greater than 2 (p < 0.05) (http://www.ebi.ac.uk/arrayexpress/experiments/E-TABM-790), suggesting that NprR has a direct or indirect effect on their transcription. Thirty-nine genes, with a relative expression ratio greater than 4, and a significance value (p) smaller than 0.01, were considered for subsequent analysis. The genes matching probes for BC2622, a macrolide glycosyltransferase, and BC3725, an exochitinase, were also investigated due to their functional similarity to the genes fulfilling these criteria. Quantitative RT-PCR confirmed that these 41 genes were at least four times up- or downregulated. Fusions to *lacZ* were constructed for nine of these genes and used to confirm that they are differentially regulated in the ∆RX mutant and wt strains (Figure S3). The expression kinetics of these genes were similar, with a sharp increase of expression after the onset of stationary phase. The final list of NprR-regulated genes is presented in Table S3. Of the 41 genes directly or indirectly regulated by NprR, 37 were down-regulated in the ∆RX strain, suggesting that NprR primarily acts as a transcriptional activator. The subcellular localizations of the products of these genes were assessed: 46% were cytoplasmic, 20% were associated with the membrane and 34% were extracellular or associated with the cell wall. The NprR-regulated genes can be distributed into four functional groups. The first group is composed of genes encoding proteins potentially involved in stress resistance: they include the genes for cytochrome P450 (BC2613), cysteine dioxygenase (BC2617), and Transporter Drug/Metabolite exporter family members (BC1063). The second group is a four-gene locus encoding the oligopeptide permease system Opp required for the import of small peptides into the cell. The third group is a five-gene locus encoding a nonribosomal peptide synthesis (NRPS) system showing similarities with the systems involved in the synthesis of secreted factors like toxins and antibiotics. The last group codes for degradative enzymes (metalloproteases, esterases and chitinases) and for proteins which can bind organic material (chitin-binding protein and collagen adhesion protein). The role of NprR in the degradation of lipids, proteins and chitin was analyzed by growing the ∆RX and wt strains on specific culture media (Figure 3A). The lipolytic, proteolytic and chitinolytic activities of the ∆RX strain were significantly lower than those of the wt strain. We monitored the expression kinetics in infected insect larvae of two NprR-regulated genes encoding degradative enzymes (BC0429 and BC2167) (Figure 3B). The two genes were specifically expressed after the insect death, from 24 h to 96 h, suggesting that *Bt* displays a necrotrophic lifestyle in the insect cadaver.

**Secreted factors regulated by NprR allow *Bt* to survive in insect cadavers.**

To identify the NprR-dependent survival factor we first tested whether this putative factor was secreted. Insect larvae were co-infected with two different ratios of wt and ∆RX strains: 90% of wt bacteria with 10% of ∆RX bacteria (90:10), and 10% of wt bacteria with 90% of ∆RX bacteria (10:90) (Figure 4A and Figure 4B). In insects infected with the ratio 10:90, the total population of the wt and the ∆RX strains decreased after 24 h. This may result from the ∆RX strain capturing NprX without being able to express NprR-regulated genes, but nevertheless removing the peptide from the environment. Consequently, the amount of signaling peptide was insufficient to activate NprR-regulated genes in the wt, resulting in clearance of both populations. Co-infection with the ratio 90:10 led to the survival of the two subpopulations during the 96 h of the experiment. In this condition, the concentration of NprX in the host was presumably sufficient to maintain the expression of the NprR-regulated genes in the wt subpopulation, and this expression allowed the survival of the ∆RX population. Therefore, the wt strain may produce secreted factors that enable the ∆RX strain to survive in the insect cadaver.

NprR-dependent extracellular factors are degradative enzymes and the factor synthesized by the NRPS system. NprA, the major degradative enzyme produced during late stationary phase, is not required for bacterial survival in insect cadaver (Figure S2). The NRPS locus consists of seven open reading frames annotated BC2450 to BC2456 in the genome of the strain *Bc* ATCC 14579 used for designing the microarrays [3]. *In silico* analysis of all available sequenced *Bt* and *Bc* genomes, including that of strain *Bt* 407 used in this study, reveals that in all cases, this locus includes only four genes (http://www.ncbi.nlm.nih.gov/bioproject/29717). Several studies suggest that these four genes (designated *krsA,B,C,E*; Figure 5A) are involved in the production of the lipopeptide kurstakin [21,22,23]. KrsE is a presumed efflux protein and KrsA, B, C are the peptide synthetase subunits. The gene *krsC* was disrupted from a wt strain by insertion of a *lacZ* gene and the survival of the mutant (∆*krsC*) in insects was monitored for 96 h (Figure 5B). A recent study shows that deletion of *krsC* abolishes the production of kurstakin in the *Bt* 407 strain [24]. The total population of the ∆*krsC* strain was maintained throughout this period as the wild type strain, indicating that the absence of kurstakin did not affect the survival of the bacteria in the insect cadaver. To determine whether kurstakin is involved in necrotrophism, we introduced a constitutive promoter upstream from these genes in the ∆RX strain. This NprR-independent expression of *krsABC* partially and significantly restored the survival of the ∆RX strain in the insect cadaver (Figure 5C). These observations indicate that the *krsABC* genes are involved in – but are not necessary for - the necrotrophic properties of *Bt*.

**Discussion**

PlcR is the main virulence regulator in *Bt* and *Bc* [10,12] and is required for the early steps of the infection process [9,25]. We show here that another quorum sensor, NprR, is active after host death and is necessary for *Bt* to survive in the insect cadaver. NprR is a pleiotropic regulator directly or indirectly affecting the expression of at least 41 genes during the stationary phase. About 30% of the NprR-regulated genes encode extracellular or cell wall-associated proteins involved in the degradation of proteins, lipids and chitin. We report that *nprA* and two other NprR-regulated genes encoding degradative enzymes were expressed after death of the host. Therefore, it is likely that these enzymes allow *Bt* to use the content of the host, indicating that *Bt* displays a necrotrophic lifestyle in the insect cadaver. This nutrient acquisition may support the developmental program of *Bt* until sporulation. The degradative enzymes may also have other functions. Insect cuticles are made of chitin filaments arranged within a protein matrix which constitutes a physical barrier to the outside environment. Degradative enzymes may degrade this barrier and facilitate spore and toxin release into the environment. Degradative enzymes may also participate in cell protection against competitors. For example, the endochitinase ChiCW was reported as having antifungal properties [26], and InhA3 (BC2984) is a member of the Immune Inhibitor A metalloprotease family, which plays a key role in the resistance to the host immune defenses by degrading antimicrobial peptides [26,27,28]. In addition, two substrate-binding proteins (BC2827 and the BC3526) may increase the efficiency of these enzymes.

A large locus of three NprR-regulated genes (*krsABC*) codes for a NRPS system involved in the synthesis of a secreted lipopeptide called kurstakin which is implicated in the necrotrophic properties, although not necessary to the survival of the bacteria in the insect cadaver. At least two suggestions could explain the function of kurstakin:

1) Lipopeptides have biosurfactant activity. Thus, the kurstakin may possibly allow *Bt* to spread across the cadaver, facilitating access to new substrates.

2) Lipopeptides are potent antimicrobials, and kurstakin has an antifungal activity [21,30,31]. Kurstakin may thus act as an antimicrobial molecule and prevent colonization and growth by competing microorganisms.

Some NprR-regulated genes encoding cytoplasmic or membrane-associated proteins may participate in the necrotrophic development of *Bt*. A putative efflux system (BC1063), two macrolide glycosyl transferases (BC2066 and BC2622) and a N-hydroxyarylamine O-acetyltransferase could be involved in resistance to antimicrobial molecules, and cytochrome P450 (BC2613) may be involved in resistance to reactive oxygen species. The membrane-associated proteins are mainly components of an oligopeptide permease system (Opp) involved in the uptake of PapR, the signaling peptide required for PlcR activation [32]. The operon encoding this Opp system is downregulated by NprR suggesting that NprR controls PlcR expression negatively through the Opp system. Interestingly, *nprA* expression was only slightly reduced in a *oppB* mutant strain suggesting that another oligopeptide permease system is involved for the uptake of NprX (Figure S4). Consequently, a down-regulation of the *opp* genes could not have a significant effect on the expression of the NprR-regulated genes. Possibly, the extracellular concentration of NprX is a signal triggering the transition from a pathogenic to a necrotrophic lifestyle. During the early stage of infection, the PlcR regulon is expressed and the extracellular concentration of NprX might be low. Indeed, recent results indicate that *nprX* transcription starts in late stationary phase [33]. In view of these data, we hypothesize that after the host death, the extracellular concentration of NprX increases, leading to the expression of NprR-regulated genes and repression of the PlcR regulon *via* the Opp transporter. These various observations indicate that the necrotrophic lifestyle of *Bt* is a complex developmental stage, not limited to simple feeding on the host contents. They also imply that the transition from a pathogenic to a necrotrophic lifestyle is associated with significant metabolic changes.

It is becoming clear that the infectious cycle of *Bt* can be divided into four distinct and sequential phases starting with toxemia caused by the Cry proteins, followed by the action of PlcR in virulence, necrotrophism and the completion of the sporulation process involving NprR, and finally the dissemination of spores (Figure 6). We describe a *nprR*-*nprX* mutant that does not develop necrotrophically and is unable to sporulate efficiently, demonstrating that the necrotrophic properties of *Bt* are essential for horizontal transmission. It is remarkable that the developmental process through the complete infection cycle in the insect host is coordinated by three regulator-signaling peptide cell-cell communication systems: PlcR-PapR, NprR-NprX and the Rap-Phr complexes which control the phosphorylation of Spo0A [34,35,36].

Members of the *Bc* group may be able to follow two different life cycles: an infectious cycle (described above) and an endosymbiotic cycle in which the bacteria live in a symbiotic relation with their invertebrate hosts [6,37]. Here, we report that *Bt* multiplies efficiently in the insect cadaver and has a genetic developmental program to live in this biotope. Consequently, we propose the existence of a strictly necrotrophic life cycle in which *Bt* colonizes a wide variety of dead insects, and uses the cadaver as a bioreactor to multiply and to produce spores and toxins.

*In silico* analysis reveals that *nprR* is found in all strains of the *Bc* group, except that in *Bc* ATCC14579 *nprR* is disrupted by a transposon [3]. The published genome sequences of *Ba* and *Bc* strains provide no evidence for any loss of genetic determinants, which might be crucial for saprophytic survival [38]. Therefore, the function of NprR is probably conserved in the *Bc* group, and it would be interesting to determine whether the necrotrophic development of *Bc* and *Ba* in their mammalian hosts requires the same quorum-sensing regulated network. It is also important to characterize kurstakin to determine how it is involved in the necrotrophic development. Moreover, the properties of this molecule may indicate possibilities for the development of phytosanitary products or adjuvants to improve both the ecological fitness and the efficacy of biopesticides.

**Materials and Methods**

Bacterial strains and growth conditions

The *Bt* strain 407 Cry^-^ is an acrystalliferous strain cured of its *cry* plasmid [39]. This strain shares high phylogenic similarity with *Bc* [40]. *Bacillus* 407 *oppB::tet, Bacillus* 407 *sigK::aphA3, Bacillus* 407 *nprRX*::*tet* (∆RX), *Bacillus* 407 *nprA::lacZ* and *Bacillus* 407 *nprRX*::*tet nprA::lacZ* mutant strains were described previously [16,20,32]. *Escherichia coli* K-12 strain TG1 was used as host for the construction of plasmids and cloning experiments. Plasmid DNA for *Bacillus* electroporation was prepared from the Dam^-^ Dcm^-^ *E. coli* strain ET12567 (Stratagene, La Jolla, CA, USA). *E. coli* and *Bt* cells were transformed by electroporation as described previously [42,44]. *E. coli* strains were grown at 37°C in Luria Broth (LB). *Bacillus* strains were grown at 30 or 37°C in LB or in HCT, a sporulation-specific medium [45]. The following concentrations of antibiotic were used for bacterial selection: 100 µg/ml ampicillin for *E. coli*; 200 µg/ml kanamycin, 10 µg/ml tetracycline, 200 µg/ml spectinomycin and 10 µg/ml erythromycin for *Bacillus*. Numbers of viable cells were counted as total colony-forming units (cfu) on LB plates. Numbers of spores were determined as heat-resistant (80°C for 12 min) cfu on LB plates.

*In vivo* experiments

Force-feeding and intrahemocelic injection experiments with *G. mellonella* were carried out as described previously [9]. LD_50_ data were analyzed using the program StatPlus 2007 of Analysoft. *Bt* cells in living and dead insects were counted as follows. For each strain, each larva was injected with 2.10^4^ bacteria and kept at 30°C for 96 h; 24 h after injection, surviving insects were eliminated. At the injection time and every 24 h for the 96 h of the experiment, two larvae were crushed and homogenized in 10 ml of physiological water and dilutions were plated onto LB agar plates containing appropriate antibiotics. To follow the spore population, bacterial colony-forming units were determined before and after treatment of the insect homogenate for 12 min at 80°C. At least four independent replicates were performed for each time and for each strain tested. *In vivo* ß-galactosidase activity was assayed from 2 ml aliquots of the insect homogenate as described previously [16]. At least three independent measurements were performed for each time and for each transcriptional fusion tested.

DNA manipulations

Chromosomal DNA was extracted from *Bt* cells using the Puregene Yeast/Bact. Kit B (QIAgen, France). Plasmid DNA was extracted from *E. coli* using QIAprep spin columns (QIAgen, France). Restriction enzymes (New England Biolabs, USA) and T4 DNA ligase (New England Biolabs, USA) were used in accordance with the manufacturer’s recommendations. Oligonucleotide primers (Table S4) were synthesized by Sigma-Proligo (Paris, France). PCRs were performed in a Applied Biosystem 2720 Thermak cycler (Applied Biosystem, USA). Amplified fragments were purified using the QIAquick PCR purification Kit (QIAgen, France). Digested DNA fragments were separated on 1 % (w/V) agarose gels after digestion and extracted from gels using the QIAquick gel extraction Kit (QIAgen, France). Nucleotide sequences were determined by Beckman Coulter Genomics (Takeley, UK)

Plasmid constructions

The plasmid pRN5101 [43] was used for homologous recombination. The low-copy-number plasmid pHT304 was used for complementation experiments with wild-type *nprR-nprX* genes under their own promoters [16]. Transcriptional fusions were constructed in pHT304-18Z [44]. All the plasmids used in this study are described in Table S5.

Construction of the *B. thuringiensis* recombinant strains

The *krsC* gene was disrupted by inserting a *lacZ* gene into the coding sequence, as described [24]. The recombinant strain, designated *Bacillus* 407 ∆*krsC*, was defective for kurstakin production [24]. The thermosensitive plasmid pRN5101ΩP*_krsABC_*::*aphA3* was used to replace the natural promoter region of the *krsABC* genes in the *Bacillus* 407 ∆RX strain by *aphA3* and its constitutive promoter. In the resulting *Bacillus* recombinant strain, the *krsABC* genes were transcribed from the *aphA3* promoter; it was designated *Bacillus* 407 ∆RX P*_aphA3_*-*krsABC*, and was resistant to kanamycin and sensitive to erythromycin.

Phenotype analysis

The methods used to study the proteolitic activity, the chitinolytic activity and the lipolytic activity have been described previously [29,45]. Strains were cultured in LB medium at 37°C until the beginning of stationary phase and 2.10^6^ bacteria were spotted onto the center of the agar plate. Plates were incubated at 37°C for 24 h to 96 h.

ß-Galactosidase assay

For *in vitro* ß-galactosidase activity measurements, *Bt* cells containing *lacZ* transcriptional fusions were cultured in LB medium at 37 °C. *In vivo* ß-galactosidase activity was assayed from 2 ml aliquots of insect homogenate (see *in vivo* experiments). ß-Galactosidase activities were measured as described previously [49]. The specific activities are expressed in units of ß-galactosidase per milligram of protein (Miller units).

Samples for microarrays and quantitative RT-PCR

Prewarmed 500 ml baffled erlenmeyer flasks with 50 ml LB medium were inoculated with 1 ml overnight cultures of *Bacillus* 407 *nprA::lacZ* or *Bacillus* 407 ΔRX *nprA::lacZ*, and incubated at 37 °C and 250 rpm. Samples for microarray analysis were taken three (t3) hours after the onset of the stationary phase. Samples were harvested as described previously [12] mixed with RLT buffer from the RNeasy midi kit (Qiagen, France) and frozen at -70 °C. After thawing samples at 37 °C for 15 min, RNA isolation, cDNA synthesis, labeling and purification were performed as described [12].

Microarray comparisons

The microarray slides were printed, prehybridized and hybridized as described previously [12], except that hybridization was extended to 17 hours. The slides were scanned on an Axon 4000B scanner (Molecular Devices). Gridding, spot annotation and calculation of raw spot intensities was done with the GenePix Pro 6.1 software (Molecular Devices). The LIMMA package [50,51,52] on the R 2.7.1 platform [53] was used for filtering, normalization and further analysis. The raw data were filtered and weighted by quality [54], and the four technical replicates on each slide were averaged to increase robustness. P-values were computed using a false discovery rate of 0.05. The analysis was based on hybridization to three slides, all employing biological replicates.

Quantitative RT-PCR

Gene expression was investigated in *Bacillus* 407 *nprA::lacZ* and *Bacillus* 407 ΔRX *nprA::lacZ*. Reverse transcription was performed according to the SuperScript III reverse transcriptase protocol from Invitrogen, but RNaseOUT was replaced with 0.1 µl SUPERase-In (Ambion). A negative control without reverse transcriptase was included. In all samples, the reaction volume was adjusted to 20 µl with DEPC-treated water (Ambion) before reverse transcription. The reaction product was diluted (1 µl in 39 µl) with water, and 8 µl applied to each well (2 µl for 5s rRNA samples). Primers were added to a final concentration of 0.56 µM. A volume of 9 µl LightCycler 480 DNA SYBR Green I Master (Roche) was added, and the volume was adjusted to 18 µl. Primers (available on request) were designed to give PCR products of around 100 bp. The reference genes, *gatB* (BC4306) and 5s rRNA, were included on every plate. The samples were analyzed on a Roche Lightcycler 480 (Roche Diagnostics GmbH, Mannheim, Germany). Cycling conditions were 95 °C for 5 minutes followed by 45 cycles at 95 °C for 10 seconds, 58 °C for 10 seconds, and 72 °C for 8 seconds. Cp values were determined using 2nd derivative max, and are averages of two technical replicates. The results were calculated by the delta-delta C_t_ approximation. The log_2_ expression ratios of *Bacillus* 407 ΔRX *nprA::lacZ* over *Bacillus* 407 *nprA::lacZ* in Table S3 are averages for three biological replicates.

**Acknowledgments**

We thank Alain Givaudan for critical reading of the manuscript and for helpful discussions. We also thank Elisabeth Guillemet for proteolytic activity assays. Thanks to Endre Anderssen, formerly part of the NTNU branch of The Norwegian Microarray Consortium, for help with the microarray analysis scripts.

**References**

1. Raymond B, Johnston PR, Nielsen-LeRoux C, Lereclus D, Crickmore N (2010) *Bacillus thuringiensis*: an impotent pathogen? Trends Microbiol 18: 189-194.

2. Toledo-Arana A, Dussurget O, Nikitas G, Sesto N, Guet-Revillet H, et al. (2009) The *Listeria* transcriptional landscape from saprophytism to virulence. Nature 459: 950-956.

3. Ivanova N, Sorokin A, Anderson I, Galleron N, Candelon B, et al. (2003) Genome sequence of *Bacillus cereus* and comparative analysis with *Bacillus anthracis*. Nature 423: 87-91.

4. Martin PAW, Travers RS (1989) Worldwide abundance and distribution of *Bacillus thuringiensis* isolates. Appl Environ Microbiol 55: 2437-2442.

5. Vilas-Boas LA, Vilas-Boas GF, Saridakis HO, Lemos MV, Lereclus D, et al. (2000) Survival and conjugation of *Bacillus thuringiensis* in a soil microcosm. FEMS Microbiol Ecol 31: 255-259.

6. Jensen GB, Hansen BM, Eilenberg J, Mahillon J (2003) The hidden lifestyles of *Bacillus cereus* and relatives. Environ Microbiol 5: 631-640.

7. Schnepf E, Crickmore N, Van Rie J, Lereclus D, Baum J, et al. (1998) *Bacillus thuringiensis* and its pesticidal crystal proteins. Microbiol Mol Biol Rev 62: 775-806.

8. Du C, Nickerson KW (1996) *Bacillus thuringiensis* HD-73 Spores Have Surface-Localized Cry1Ac Toxin: Physiological and Pathogenic Consequences. Appl Environ Microbiol 62: 3722-3726.

9. Salamitou S, Ramisse F, Brehélin M, Bourguet D, Gilois N, et al. (2000) The *plcR* regulon is involved in the opportunistic properties of *Bacillus thuringiensis* and *Bacillus cereus* in mice and insects. Microbiology 146: 2825-2832.

10. Agaisse H, Gominet M, Økstad OA, Kolstø AB, Lereclus D (1999) PlcR is a pleiotropic regulator of extracellular virulence factor gene expression in *Bacillus thuringiensis*. Mol Microbiol 32: 1043-1053.

11. Slamti L, Lereclus D (2002) A cell-cell signaling peptide activates the PlcR virulence regulon in bacteria of the *Bacillus cereus* group. EMBO J 21: 4550-4559.

12. Gohar M, Faegri K, Perchat S, Ravnum S, Økstad OA, et al. (2008) The PlcR virulence regulon of *Bacillus cereus*. PLoS ONE 3: e2793.

13. Gohar M, Økstad OA, Gilois N, Sanchis V, Kolstø A-B, et al. (2002) Two-dimensional electrophoresis analysis of the extracellular proteome of *Bacillus cereus* reveals the importance of the PlcR regulon. Proteomics 2: 784-791.

14. Lereclus D, Agaisse H, Grandvalet C, Salamitou S, Gominet M (2000) Regulation of toxin and virulence gene transcription in *Bacillus thuringiensis*. Int J Med Microbiol 290: 295-299.

15. Chitlaru T, Gat O, Gozlan Y, Ariel N, Shafferman A (2006) Differential proteomic analysis of the *Bacillus anthracis* secretome: distinct plasmid and chromosome CO2-dependent cross talk mechanisms modulate extracellular proteolytic activities. J Bacteriol 188: 3551-3571.

16. Perchat S, Dubois T, Zouhir S, Gominet M, Poncet S, et al. (2011) A cell-cell communication system regulates protease production during sporulation in bacteria of the Bacillus cereus group. Mol Microbiol 82: 619-633.

17. Chung MC, Popova TG, Millis BA, Mukherjee DV, Zhou W, et al. (2006) Secreted neutral metalloproteases of *Bacillus anthracis* as candidate pathogenic factors. J Biol Chem 281: 31408-31418.

18. Hajaij-Ellouze M, Fedhila S, Lereclus D, Nielsen-LeRoux C (2006) The enhancin-like metalloprotease from the *Bacillus cereus* group is regulated by the pleiotropic transcriptional activator PlcR but is not essential for larvicidal activity. FEMS Microbiol Lett 260: 9-16.

19. Hilbert DW, Piggot PJ (2004) Compartmentalization of gene expression during *Bacillus subtilis* spore formation. Microbiol Mol Biol Rev 68: 234-262.

20. Bravo A, Agaisse H, Salamitou S, Lereclus D (1996) Analysis of *cryIAa* expression in *sigE* and *sigK* mutants of *Bacillus thuringiensis*. Mol Gen Genet 250: 734-741.

21. Hathout Y, Ho YP, Ryzhov V, Demirev P, Fenselau C (2000) Kurstakins: a new class of lipopeptides isolated from *Bacillus thuringiensis*. J Nat Prod 63: 1492-1496.

22. Bumpus SB, Evans BS, Thomas PM, Ntai I, Kelleher NL (2009) A proteomics approach to discovering natural products and their biosynthetic pathways. Nat Biotechnol 27: 951-956.

23. Abderrahmani A, Tapi A, Nateche F, Chollet M, Leclere V, et al. (2011) Bioinformatics and molecular approaches to detect NRPS genes involved in the biosynthesis of kurstakin from *Bacillus thuringiensis*. Appl Microbiol Biotechnol.

24. Gélis-Jeanvoine S, Canette A, Gohar M, Caradec T, Lemy C, et al. (2016) Genetic and functional analyses of *krs*, a locus encoding kurstakin, a lipopeptide produced by *Bacillus thuringiensis.* Res Microbiol. doi: 10.1016/j.resmic.2016.06.002.

25. Fedhila S, Gohar M, Slamti L, Nel P, Lereclus D (2003) The *Bacillus thuringiensis* PlcR-regulated gene *inhA2* is necessary, but not sufficient, for virulence. J Bacteriol 185: 2820-2825.

26. Huang CJ, Wang TK, Chung SC, Chen CY (2005) Identification of an antifungal chitinase from a potential biocontrol agent, *Bacillus cereus* 28-9. J Biochem Mol Biol 38: 82-88.

27. Dalhammar G, Steiner H (1984) Characterization of inhibitor A, a protease from *Bacillus thuringiensis* which degrades attacins and cecropins, two classes of antibacterial proteins in insects. Eur J Biochem 139: 247-252.

28. Ramarao N, Lereclus D (2005) The InhA1 metalloprotease allows spores of the *B. cereus* group to escape macrophages. Cell Microbiol 7: 1357-1364.

29. Guillemet E, Cadot C, Tran SL, Guinebretiere MH, Lereclus D, et al. (2010) The InhA metalloproteases of *Bacillus cereus* contribute concomitantly to virulence. J Bacteriol 192: 286-294.

30. Carrillo C, Teruel JA, Aranda FJ, Ortiz A (2003) Molecular mechanism of membrane permeabilization by the peptide antibiotic surfactin. Biochim Biophys Acta 1611: 91-97.

31. Stein T (2005) *Bacillus subtilis* antibiotics: structures, syntheses and specific functions. Mol Microbiol 56: 845-857.

32. Gominet M, Slamti L, Gilois N, Rose M, Lereclus D (2001) Oligopeptide permease is required for expression of the *Bacillus thuringiensis* PlcR regulon and for virulence. Mol Microbiol 40: 963-975.

33. Dubois T, Perchat S, Verplaetse E, Gominet M, Lemy C, et al. (2013) Activity of the *Bacillus thuringiensis* NprR-NprX cell-cell communication system is co-ordinated to the physiological stage through a complex transcriptional regulation. Mol Microbiol 88: 48-63.

34. Perego M, Hoch JA (1996) Protein aspartate phosphatases control the output of two-component signal transduction systems. Trends Genet 12: 97-101.

35. Lazazzera BA, Grossman AD (1998) The ins and outs of peptide signaling. Trends Microbiol 6: 288-294.

36. Bongiorni C, Stoessel R, Shoemaker D, Perego M (2006) Rap phosphatase of virulence plasmid pXO1 inhibits *Bacillus anthracis* sporulation. J Bacteriol 188: 487-498.

37. Margulis L, Jorgensen JZ, Dolan S, Kolchinsky R, Rainey FA, et al. (1998) The Arthromitus stage of *Bacillus cereus*: intestinal symbionts of animals. Proc Natl Acad Sci U S A 95: 1236-1241.

38. Saile E, Koehler TM (2006) Bacillus anthracis multiplication, persistence, and genetic exchange in the rhizosphere of grass plants. Appl Environ Microbiol 72: 3168-3174.

39. Lereclus D, Arantes O, Chaufaux J, Lecadet M-M (1989) Transformation and expression of a cloned ∂-endotoxin gene in *Bacillus thuringiensis*. FEMS Microbiol Lett 60: 211-218.

40. Kolstø AB, Tourasse NJ, Økstad OA (2009) What sets *Bacillus anthracis* apart from other *Bacillus* species? Annu Rev Microbiol 63: 451-476.

41. Dower WJ, Miller JF, Ragsdale CW (1988) High efficiency transformation of *E. coli* by high voltage electroporation. Nucleic Acids Res 16: 6127-6145.

42. Lecadet MM, Blondel MO, Ribier J (1980) Generalized transduction in *Bacillus thuringiensis* var. *berliner* 1715, using bacteriophage CP54 Ber. J Gen Microbiol 121: 203-212.

43. Lereclus D, Vallade M, Chaufaux J, Arantes O, Rambaud S (1992) Expansion of insecticidal host range of *Bacillus thuringiensis* by in vivo genetic recombination. Bio/Technology 10: 418-421.

44. Agaisse H, Lereclus D (1994) Structural and functional analysis of the promoter region involved in full expression of the *cryIIIA* toxin gene of *Bacillus thuringiensis*. Mol Microbiol 13: 97-107.

45. de Barjac H, Cosmao-Dumanoir V (1975) Interest of some additional biochemical tests for the classification of "Bacillus" (author's transl). Ann Microbiol (Paris) 126: 83-95.

46. Bouillaut L, Ramarao N, Buisson C, Gilois N, Gohar M, et al. (2005) FlhA influences *Bacillus thuringiensis* PlcR-regulated gene transcription, protein production, and virulence. Appl Environ Microbiol 71: 8903-8910.

47. Smyth GK (2004) Linear models and empirical bayes methods for assessing differential expression in microarray experiments. Stat Appl Genet Mol Biol 3: Article3.

48. Smyth GK, Speed T (2003) Normalization of cDNA microarray data. Methods 31: 265-273.

49. Smyth GK (2005) Limma: Linear models for microarray data. In: Gentleman VCR, Dudoit S, Irizarry R, Huber W, editors. Bioinformatics and Computational Biology Solutions using R and Bioconductor. New York: Springer. pp. 397-420.

50. R-Development-Core-Team (2005) R: A language and environment for statistical computing. Vienna (Austria): R Foundation for Statistical Computing. Available: http://www.R-project.org.

51. Bruland T, Anderssen E, Doseth B, Bergum H, Beisvag V, et al. (2007) Optimization of cDNA microarrays procedures using criteria that do not rely on external standards. BMC Genomics 8: 377.

**Figure legends**

**Figure 1. *mpbE* and *nprA* are sequentially activated during infection.** (A) Correlation between the β-galactosidase activity obtained with the P*_aphA3_*-*lacZ* fusion and the number of bacteria of the reporter strain 407 p*apha3*’Z in infected insects. The activity of the *aphA3* promoter remains constant over time in insects such that the P*_aphA3_*-*lacZ* fusion can be used as an *in vivo* reference standard. (B) Expression of the *mpbE* and *nprA* genes *in vivo*. Each point is the log-transformed ratio of the β-galactosidase activity obtained with the 407 p*nprA’*Z and 407 p*mpbE*’Z strains to that with the control strain 407 p*aphA3*’Z. The black arrow indicates that assays were carried out with dead insects from 24 h post infection until the end of the experiment. Data are averages of at least three independent experiments (error bars are SEM from mean values).

**Figure 2. NprR allows *Bt* to survive in insect cadavers by a process independent of sporulation.** (A) Comparison of the wt and ∆RX strain survival in the insect host. The survival defect of the mutant is genetically complemented by pHT304-RX. (B) Effect of the *nprR-nprX* deletion on sporulation. (C) Comparison of the wt and ∆*sigK* strain survival in the insect host. Data are averages of at least four independent experiments (error bars are SEM from mean values).

**Figure 3. NprR is involved in the necrotrophic development of *Bt* in the insect cadaver.** (A) Growth of the wt and ∆RX strains on specific culture media. The lipolytic activity was assayed on TS medium supplemented with Tween 80. Wild-type strain colonies were surrounded by a precipitate of oleic acid (a Tween 80 degradation product) whereas no precipitate was detected around the mutant strain colonies. The proteolytic and chitinolytic activities were assayed on HCT medium supplemented with cow’s milk and on chitin medium, respectively. In both conditions, the wt strain was surrounded by a degradation ring whereas no such rings were detected for the mutant. (B) The genes BC0429 and BC2167 were specifically expressed after the insect death. Each point is the log-transformed ratio of the β-galactosidase activity obtained with the 407 pBC0429’Z and 407 pBC2167’Z strains to that with the control strain 407 p*aphaA3*’Z. The growth kinetics of both reporter strains are presented in Fig. S1B. Data are averages of at least three independent experiments (error bars are SEM from mean values).

**Figure 4. A NprR-regulated secreted factor allows *Bt* to survive in the insect host.** Insect larvae were co-infected with both wt and ∆RX strains mixed at two different ratios: (A) 90% wt bacteria with 10% ∆RX bacteria (90:10) and (B) 10% wt bacteria with 90% ∆RX bacteria (10:90). Data are averages of at least four independent experiments (error bars are SEM from mean values).

**Figure 5. The *krs* operon involved in the production of kurstakin is implicated in - but is not essential for - the necrotrophic lifestyle of *Bt*.** (A) Genetic organization of the *krsEABC* locus in *Bc* ATCC14579 and *Bt* strain 407. *krsE* encodes a putative efflux protein. *krsA*, *krsB* and *krsC* encode non ribosomal peptide synthetases KrsA, KrsB and KrsC, respectively. Immediately downstream is a gene encoding a phosphopanthetheinyl transferase (ppant) and a gene encoding a protein with a thioesterase domain; both these genes are involved in kurstakin synthesis. (B) Comparaison of the wt and ∆*krsC* strain survival in the insect host. (C) The constitutive expression of the *krs* operon in the ∆RX strain restored the survival of this mutant.

**Figure 6. Sequential expression of master regulators during the infectious cycle of *Bt.*** (1) Toxins and spores reach the intestinal epithelium. The toxins induce cell lysis and create favorable conditions for spore germination. The resulting vegetative bacteria adhere to cells weakened by toxins and can grow. (2) The bacteria produce PlcR-regulated virulence factors which attack the intestinal barrier. This event allows the bacteria to penetrate into deeper tissues, such that they can multiply in body fluids and cause host death. (3) The bacteria shift from a virulent lifestyle to a necrotrophic lifestyle and change their gene expression pattern. The pleiotropic regulator NprR activates the transcription of genes encoding proteins involved in food supply, stress resistance, protection against competitors and kurstakin synthesis. These factors increase bacterial fitness in the host cadaver, allowing the bacteria to produce Cry toxins (orange diamonds) and to pursue their developmental program until sporulation and production of resistant spores. (4) Spores and toxins disseminate in the environment through various abiotic (wind, rain) and biotic (parasites, predators) mechanisms.

**Figure S1. Growth kinetics of reporter strains.** (A) Total population of the 407 p*aphA3*’Z, 407 p*nprA’*Z and 407 p*mpbE*’Z strains in the insect larvae. (B) Total population of the 407 pBC0429’Z and 407 pBC2167’Z strains in the insect larvae. Data are averages of at least three independent experiments (error bars are SEM from mean values).

**Figure S2. *nprA* is not required for the necrotrophic lifestyle of *Bt*.** The total population of the ∆*nprA* strain was similar to that of the wt strain throughout the experiment indicating that *nprA* is not required for *Bt* to survive in the insect host. Data are averages of at least four independent experiments (error bars are SEM from mean values).

**Figure S3. Differential expression of nine genes was confirmed with *lacZ* fusions.** Fusions of the promoter region of the genes tested to a *lacZ* reporter on the plasmid pHT304-18Z were introduced into the wt (circles) and the *∆*RX strains (squares), and expression was measured. Time on the x-axis is given relative to the transition to stationary phase (t0). β-Galactosidase activity in Miller units (MU) is plotted on the y-axis. Each assay was repeated at least twice independently and a representative graph is shown for each experiment.

**Figure S4. Expression of the P*_nprA_*-*lacZ* chromosomal fusions into the wt (circles) and the *∆oppB* strains (squares).** Time on the x-axis is given relative to the transition to stationary phase (t0). β-Galactosidase activity in Miller units (MU) is plotted on the y-axis. Assays were repeated at least three times independently and a representative graph is shown.

**Table S1. NprR is not involved in the pathogenicity of *Bt*.**

**Table S2. The ∆RX strain is unable to sporulate in the insect host.** These experiments were done at 30°C. The percentages were calculated as 100 × the ratio between heat-resistant spores ml^−1^ and viable cells ml^−1^. For both strains, the OD_600_ at t0 in HCT medium was 2.2 ± 0.1 and that in LB was 2.6 ± 0.1. n is the number of independent sporulation efficiency measurements. Results are given as mean ± SEM.

**Table S3. NprR is a pleiotropic regulator.** The NprR-regulated genes can be classified into four functional groups encoding: stress resistance proteins (in purple); an oligopeptide permease (in green); a NRPS system (in orange); and food supply proteins (in blue). This table includes genes more than four times differentially regulated in ΔRX strain relative to the wt strain at t3 in LB medium, as confirmed by qRT-PCR (expression ratios). Locus tags for the *Bc* ATCC 14579 microarray probes are listed.

**Table S4. Primers used for construction of recombinant strains and transcriptional fusions.** Restriction sites are underlined.

**Table S5. Plasmids used in this study.**


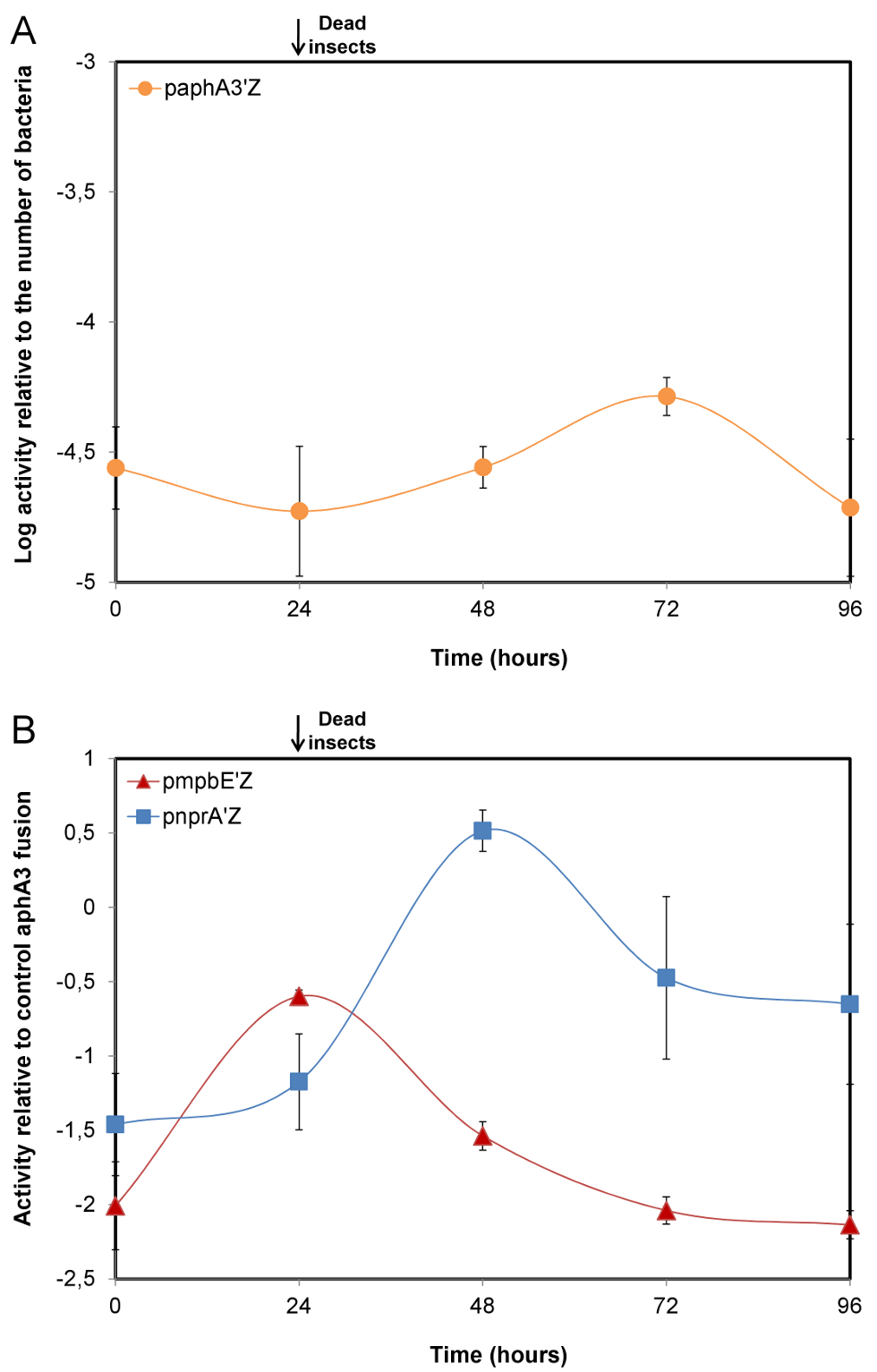


Figure 1


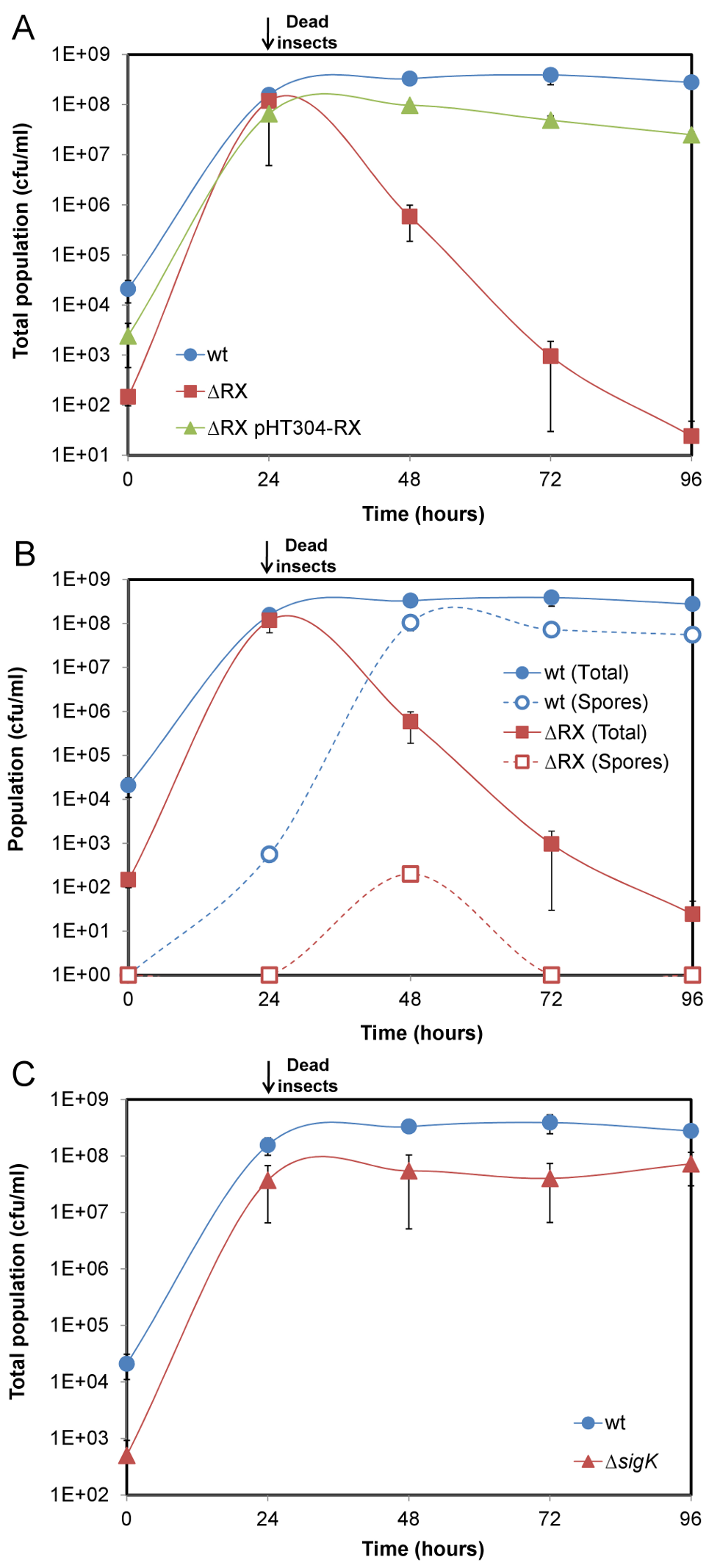


Figure 2


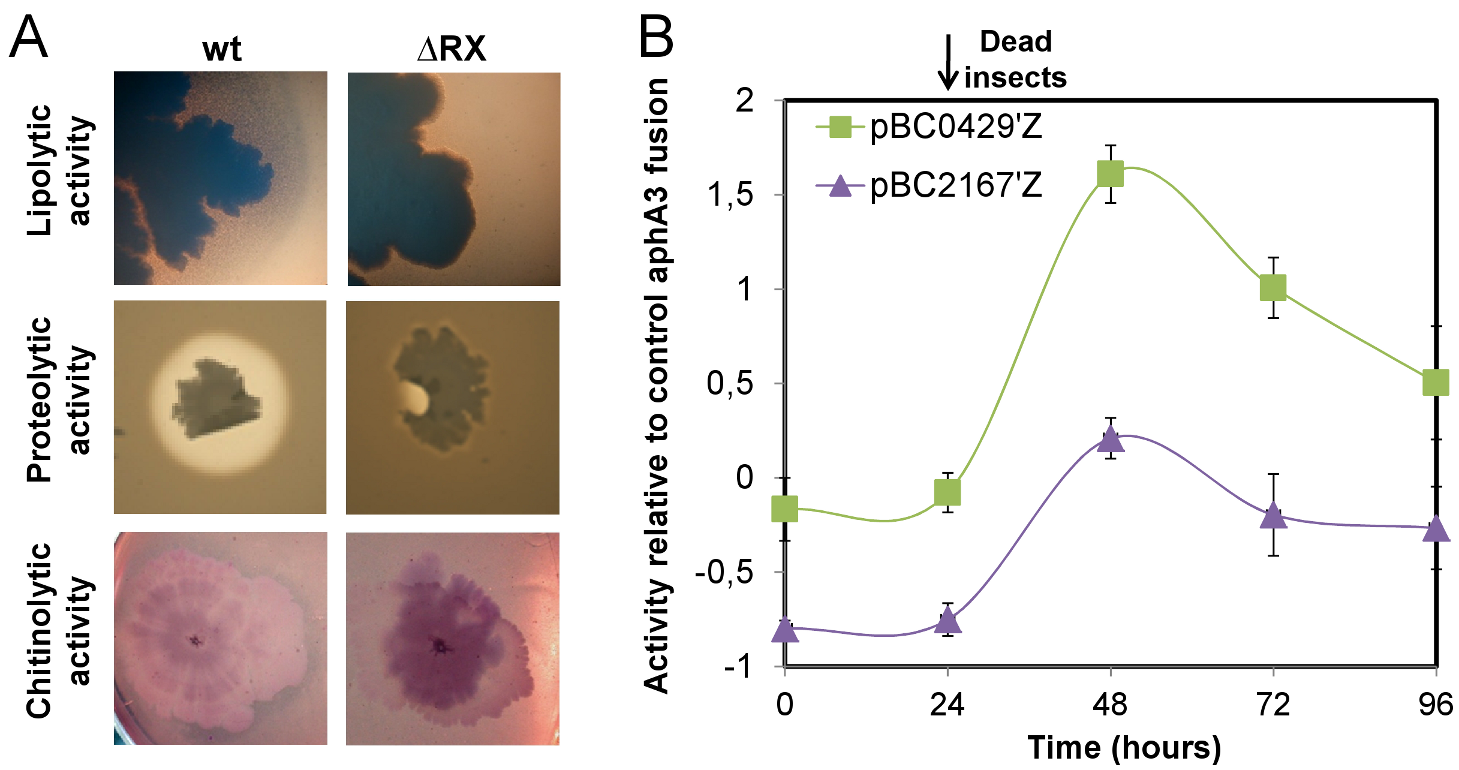


Figure 3


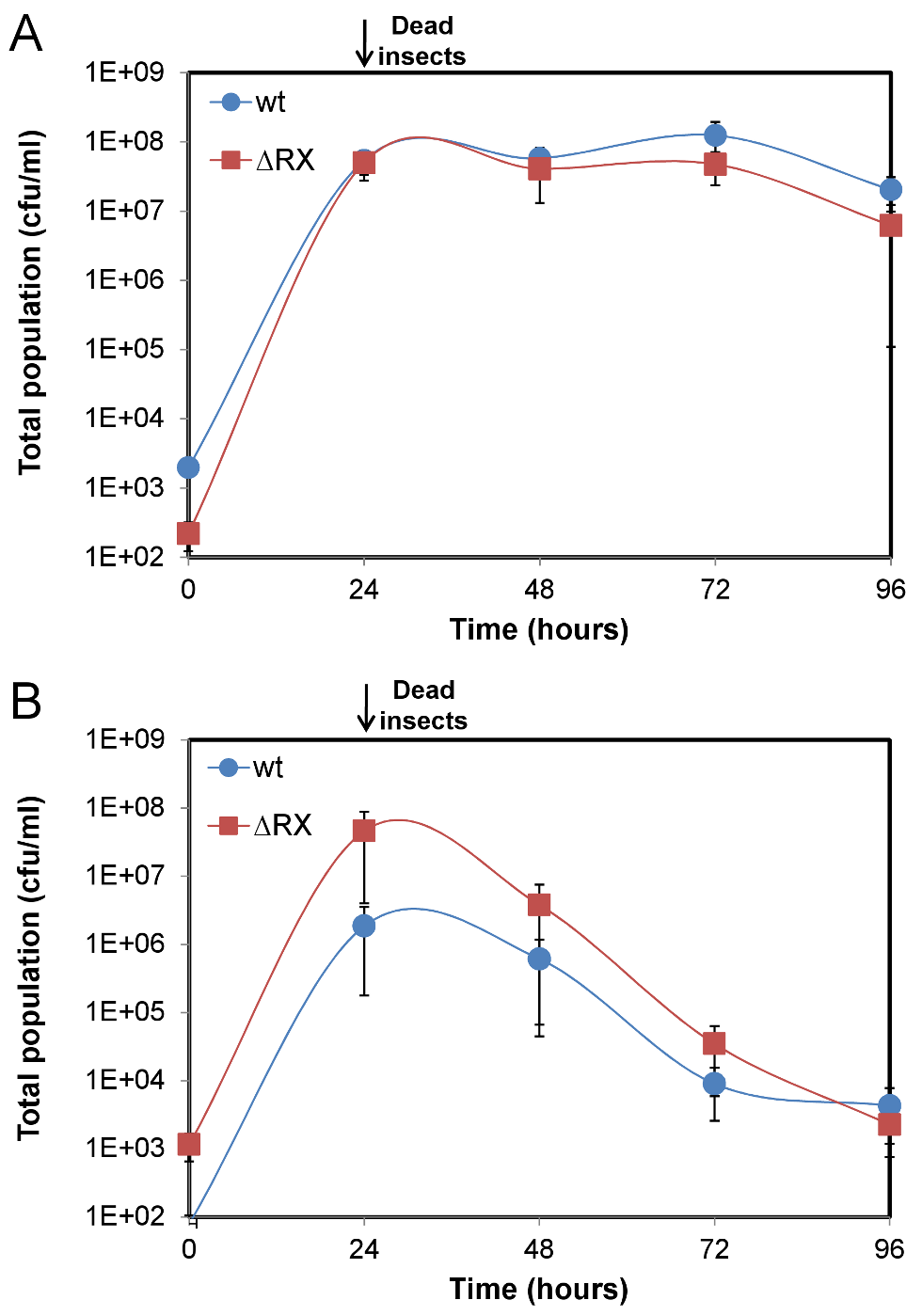


Figure 4


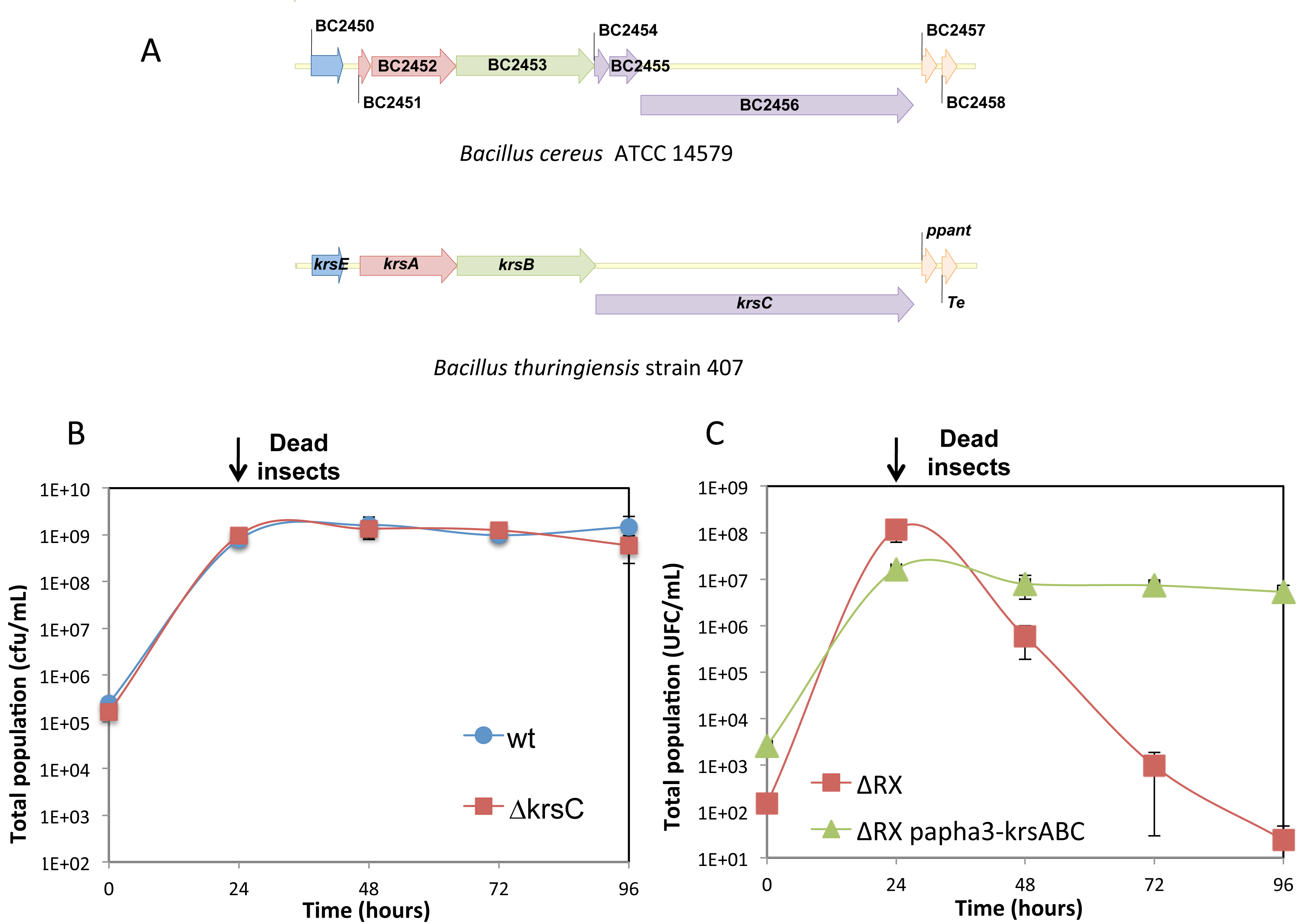


Figure 5


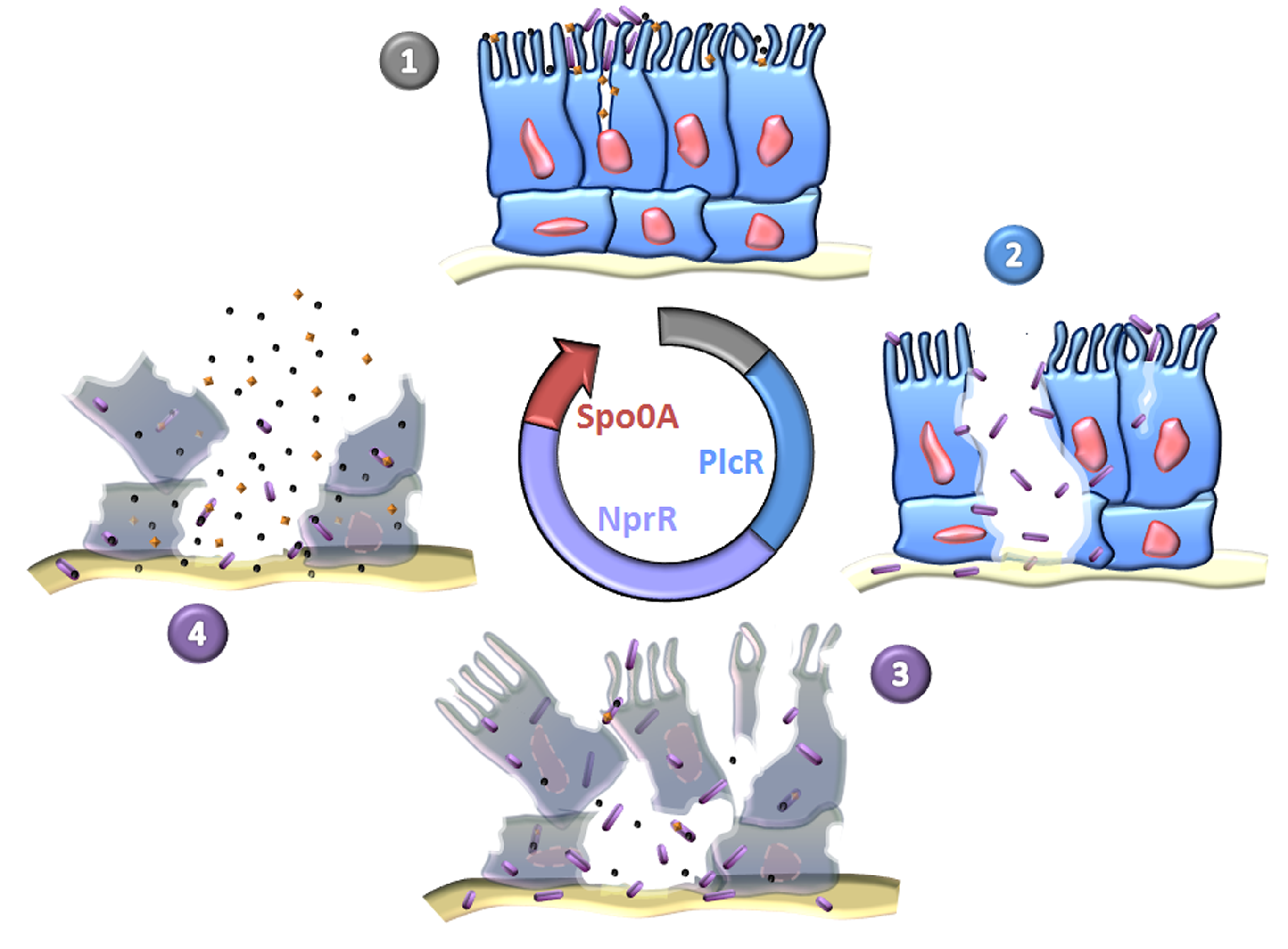


Figure 6


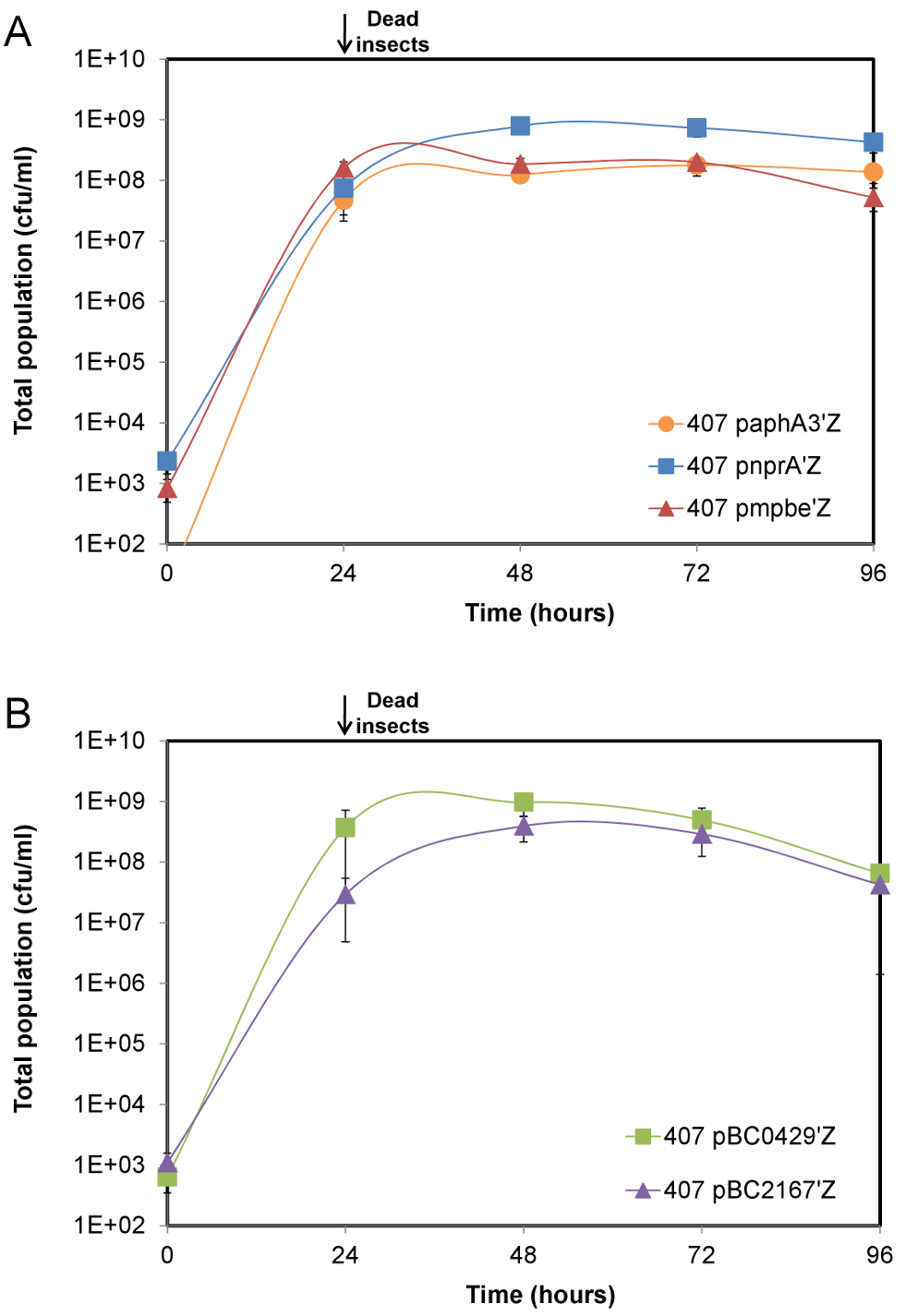


Figure S1


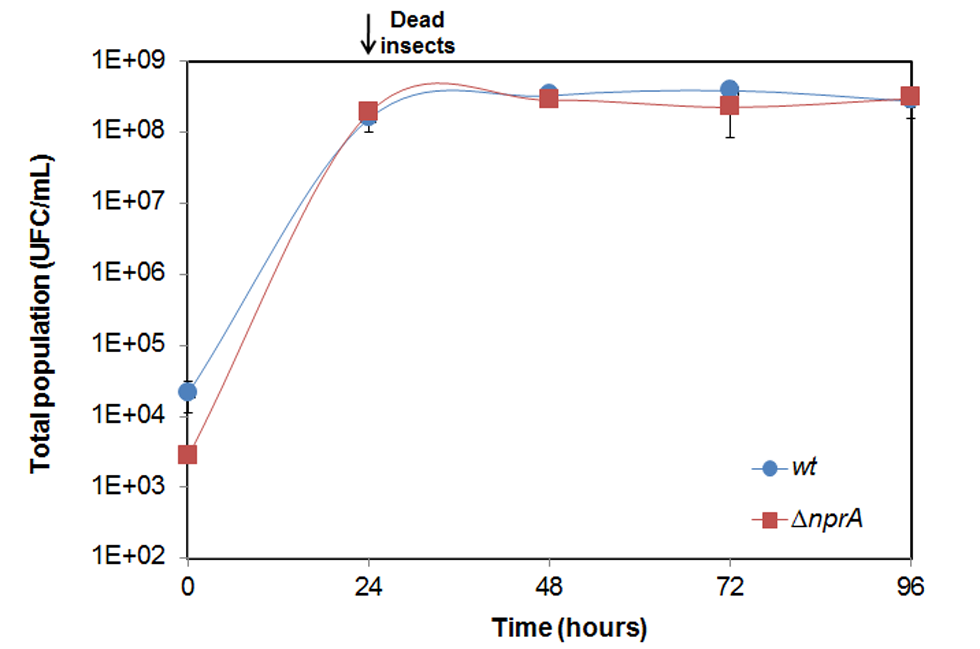


Figure S2


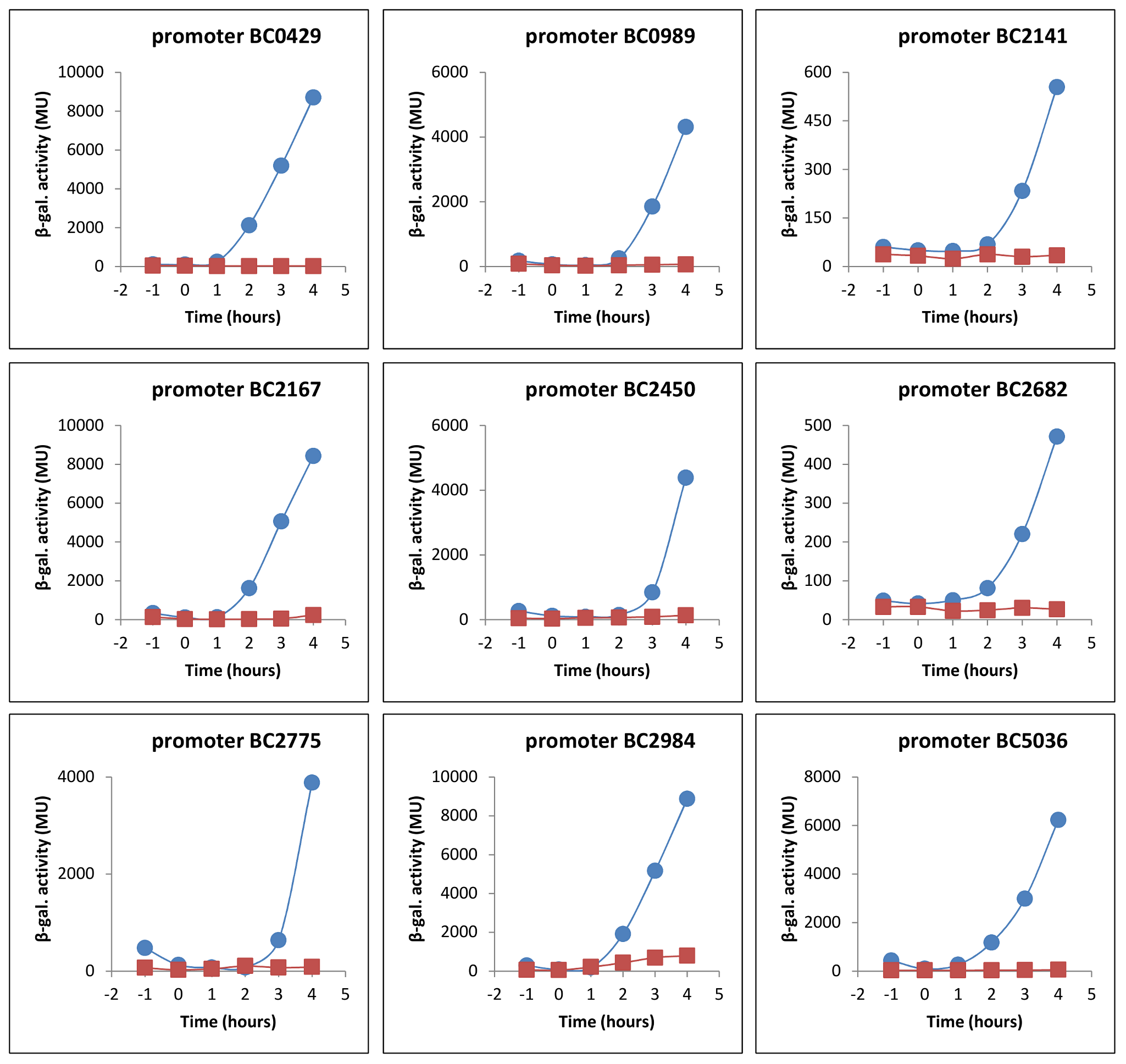


Figure S3


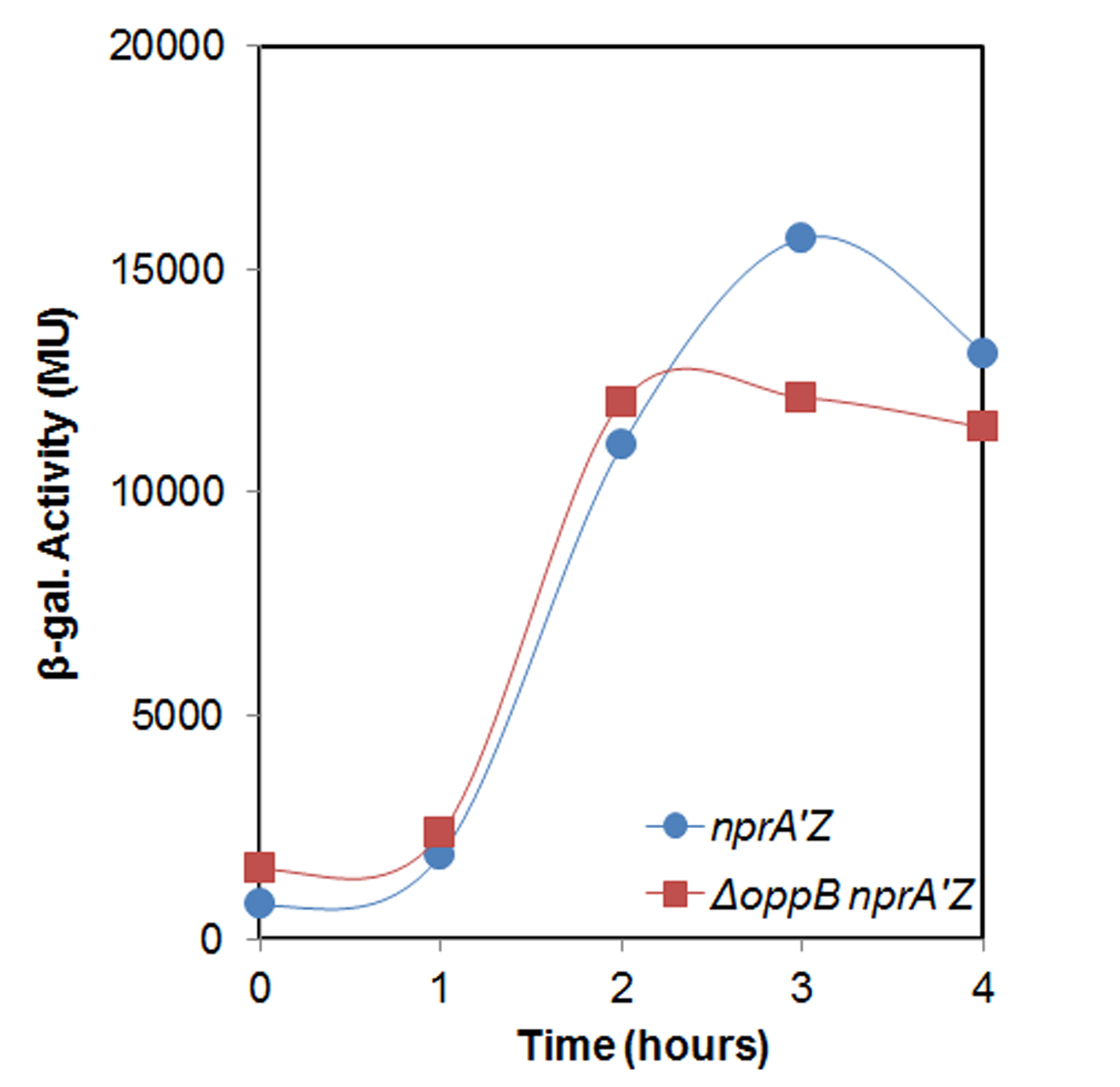


Figure S4

| **Strains** | **Force-feeding** | | **Injection** | |
| --- | --- | --- | --- | --- |
|  | **LD_50_** | **CI (95%)** | **LD_50_** | **CI (95%)** |
| wt | 7.0 x 10^6^ | 5.1 x 10^6^ – 9.0 x 10^6^ | 2.8 x 10^3^ | 2.3 x 10^3^ – 3.3 x 10^3^ |
| ∆RX | 8.2 x 10^6^ | 5.9 x 10^6^ – 10.6 x 10^6^ | 2.8 x 10^3^ | 2.2 x 10^3^ – 3.3 x 10^3^ |

CI : confidence intervals

Table S1

| **strains** | **Medium** | **Time (hours)** | **Viable counts ml−1** | **Heat-resistant spores ml−1** | **% spores** | **n** |
| --- | --- | --- | --- | --- | --- | --- |
| wt | HCT | 48 | 4.49E+08 ± 3.82E+07 | 3.80E+08 ± 3.89E+07 | 85 ± 2 | 3 |
|  | LB | 72 | 2.69E+08 ± 1.81E+07 | 1.58E+08 ± 1.13E+07 | 59 ± 1 | 7 |
|  | Insects | 0 | 2.09E+04 ± 9.85E+03 | 0.00E+00 ± 0.00E+00 | 00 ± 0 | 7 |
|  |  | 24 | 1.56E+08 ± 5.33E+07 | 5.57E+02 ± 1.68E+02 | 00 ± 0 | 7 |
|  |  | 48 | 3.31E+08 ± 5.13E+07 | 1.05E+08 ± 3.66E+07 | 32 ± 12 | 7 |
|  |  | 72 | 3.91E+08 ± 1.43E+08 | 7.24E+07 ± 2.36E+07 | 19 ± 8 | 6 |
|  |  | 96 | 2.78E+08 ± 5.22E+07 | 5.53E+07 ± 1.89E+07 | 20 ± 6 | 7 |
| ΔRX | HCT | 48 | 4.56E+08 ± 1.88E+07 | 3.46E+08 ± 7.78E+06 | 76 ± 2 | 5 |
|  | LB | 72 | 1.77E+08 ± 2.42E+07 | 8.30E+07 ± 1.43E+07 | 47 ± 2 | 7 |
|  | Insects | 0 | 1.47E+02 ± 4.99E+01 | 0.00E+00 ± 0.00E+00 | 00 ± 0 | 5 |
|  |  | 24 | 1.17E+08 ± 5.60E+07 | 1.00E+00 ± 1.00E+00 | 00 ± 0 | 5 |
|  |  | 48 | 5.83E+05 ± 3.96E+05 | 2.00E+02 ± 2.00E+02 | 00 ± 0 | 5 |
|  |  | 72 | 9.54E+02 ± 9.24E+02 | 0.00E+00 ± 0.00E+00 | 00 ± 0 | 5 |
|  |  | 96 | 2.42E+01 ± 2.40E+01 | 0.00E+00 ± 0.00E+00 | 00 ± 0 | 5 |

Table S2

| **Locus tag** | **Name** | **predicted localisation** | **expression ratios** |
| --- | --- | --- | --- |
| BC1061 | hypothetical protein | cytoplasmic | 0,034 |
| BC1062 | 3-oxoacyl-[acyl-carrier protein] reductase | cytoplasmic | 0,024 |
| BC1063 | transporter. Drug/Metabolite Exporter family | membrane | 0,177 |
| BC2066 | macrolide glycosyl transferase | cytoplasmic | 0,008 |
| BC2622 | macrolide glycosyltransferase | cytoplasmic | 0,130 |
| BC2617 | cysteine dioxygenase | cytoplasmic | 0,023 |
| BC2140 | N-hydroxyarylamine O-acetyltransferase | cytoplasmic | 0,008 |
| BC2613 | cytochrome P450 | cytoplasmic | 0,056 |
| BA3286 | peroxydase | extracellular | 0,029 |
| BC1180 | oligopeptide ABC transporter permease protein OppB | membrane | 11,472 |
| BC1181 | oligopeptide ABC transporter.permease protein OppC | membrane | 12,996 |
| BC1182 | oligopeptide ABC transporter ATP-binding protein OppD | membrane | 15,207 |
| BC1183 | oligopeptide ABC transporter ATP-binding protein OppF | membrane | 11,917 |
| BC2450 | macrolide-efflux protein | membrane | 0,012 |
| BC2451 | peptide synthetase | cytoplasmic | 0,008 |
| BC2452 | peptide synthetase | cytoplasmic | 0,012 |
| BC2453 | peptide synthetase | cytoplasmic | 0,010 |
| BC2454 | peptide synthetase | cytoplasmic | 0,014 |
| BC2455 | peptide synthetase | cytoplasmic | 0,018 |
| BC2456 | peptide synthetase | cytoplasmic | 0,017 |
| BC0602 | neutral protease NprA | extracellular | 0,006 |
| BC2167 | neutral protease | extracellular | 0,006 |
| BC2984 | immune inhibitor A precursor InhA3 | extracellular | 0,103 |
| BC5036 | neutral protease | extracellular | 0,005 |
| Bant_01003380 | esterase/Lipase | cytoplasmic | 0,197 |
| BC2141 | lipase | extracellular | 0,045 |
| BC2743 | carboxylesterase | cytoplasmic | 0,030 |
| BC0429 | endochitinase ChiCW | extracellular | 0,011 |
| BC2682 | chitosanase | extracellular | 0,004 |
| BC3725 | exochitinase ChiCH | extracellular | 0,031 |
| BC2827 | chitin-binding protein | extracellular | 0,059 |
| BC3526 | collagen adhesion protein | cell wall | 0,064 |
| BC1697 | asparagine synthetase. glutamine-hydrolyzing | cytoplasmic | 0,029 |
| BC1848 | prophage helix-turn-helix protein | cytoplasmic | 0,123 |
| BC1997 | intracellular serine protease | cytoplasmic | 0,117 |
| BC0989 | hypothetical protein | extracellular | 0,001 |
| BC1241 | hypothetical protein | cytoplasmic | 0,118 |
| BC1243 | hypothetical protein | membrane | 0,134 |
| BC1244 | hypothetical protein | membrane | 0,226 |
| BC2775 | hypothetical protein | extracellular | 0,001 |
| BC5360 | hypothetical protein | extracellular | 0,046 |

Table S3

| **Primer name** | **Primer sequences** | **Restriction site** |
| --- | --- | --- |
| **NRPS C1** | CGCGGATCCGCAAAGCGTGGGTTAGTAACTA | *Bam*HI |
| **NRPS C2** | AACTGCAGATAGATGGCTTTAGCCATAAGGAT | *Pst*I |
| **NRPS C3** | CCGCGCATGCACCTTATACGCAAAAGCGTTTA | *Sph*I |
| **NRPS C4** | CCCAAGCTTAGATACAAGCATCAGCCCACT | *Hind*III |
| **pApha3-1** | GCATGCCTGCAGGTGATAAACC | *Pst*I |
| **pApha3-2** | GCTCTAGACAATTCCGGTGATATTCTCATTTTACCC | *Xba*I |
| **Kana Cfwd** | AACTGCAGCGAACCATTTGAGGTGATAG | *Pst*I |
| **Kana Crev** | CCGCGCATGCTAAATCTAGGTACTAAAACAATTCATCC | *Sph*I |
| **BC0429F** | CGCGGATCCAGTGTGAATTTTTGAGACCTCATAGC | *Bam*HI |
| **BC0429R** | CCCAAGCTTCCATTTCTGGTGGAATCATTCCCGC | *Hin*dIII |
| **BC0989F** | AACTGCAGTTAGTGCAAATATGTTATTCGGATC | *Pst*I |
| **BC0989R** | GCTCTAGATGCAATACCTGTTGCTAACGCTCC | *Xba*I |
| **BC2141F** | AACTGCAGAATTTTCTTCTGCTCACCGATAG | *Pst*I |
| **BC2141R** | GCTCTAGACCATTCTCCAGGTGTGAATACTTCTG | *Xba*I |
| **BC2167F** | AACTGCAGCACATAATTCTGTGAAAATACATTCG | *Pst*I |
| **BC2167R** | GCTCTAGAAATCGTCCCCATTACTAACGGC | *Xba*I |
| **BC2450F** | AACTGCAGCTTTTTGTTTAGTAGGATTTATAGTCTCT | *Pst*I |
| **BC2450R** | GCTCTAGACCAAGCATAAGCAATGAATAGG | *Xba*I |
| **BC2682F** | AACTGCAGGAATTGCCTTAATTTCATTTAG | *Pst*I |
| **BC2682R** | GCTCTAGAATTTTGTATTGAATTGTCCGTTAC | *Xba*I |
| **BC2775F** | AACTGCAGTTCACCTGATGGGTATTCTGAATTAG | *Pst*I |
| **BC2775R** | GCTCTAGAGCACTTACCGCACCTAGTCCTCC | *Xba*I |
| **BC2984F** | CGGGATCCAGCATGCGCTGCGTGACCTCC | *Bam*HI |
| **BC2984R** | CCCAAGCTTGTAACAGAGAATCACGCTATA | *Hin*dIII |
| **BC5036F** | GCTCTAGATAACAGTTAACGCTAATGCTGATG | *Xba*I |
| **BC5036R** | AACTGCAGCATAATTTAGGCGATGAAACGC | *Pst*I |

Table S4

| **Plasmid name** | **Plasmid features** |
| --- | --- |
| pRN5101ΩP*_krsABC_*::*aphA3* | 5' and 3' regions of the NRPS promoter region were amplified using primers NRPS C1/NRPS C2 and NRPS C3/NRPS C4, respectively, and 407 chromosomal DNA as template. The 5' end was purified as a *Bam*HI-*Pst*I fragment and the 3' end as a *SphI*–*Hin*dIII fragment. A 1.3 kb fragment containing the kanamycine resistance gene with *aphA3* promoter but without it final stern-loop structure was amplified by PCR using primers Kana Cfwd / Kana Crev from pDG783 (Guérout-Fleury *et* *al.*, 1995), purified as a *Pst*I-*Sph*I fragment and inserted with the 5' and 3' parts of the NRPS promoter region between the *Hin*dIII and *Bam*HI sites of pRN5101. |
| pRN5101Ω*nprA*::*lacZ* | This plasmid was previously described (Perchat *et al*., 2011). |
| pHT304-RX | This plasmid was previously described (Perchat *et al*., 2011). |
| paphA3*’*Z | The promoter region of *apha3* gene was amplified using primers pApha3-1/ pApha3-2 with pDG783 as template and inserted between the *Pst*I and *Xba*I sites of pHT304.18Ω*lacZ*. This results in the creation of a transcriptional fusion between *apha3* promoter and *lacZ* gene. |
| pnprA’Z | This plasmid was previously described as pA1’Z (Perchat *et al*., 2011). |
| pmpbE’Z | This plasmid was previously described as pHT304mpbE’Z (Hajaij-Ellouze *et al*., 2006). |
| pBC0429’*Z* | The promoter region of BC0429 gene was amplified using primers BC0429F/ BC0429R with 407 chromosomal DNA as template and inserted between the *Hind*III and *Bam*HI sites of pHT304.18Ω*lacZ*. This results in the creation of a transcriptional fusion between BC0429 promoter and *lacZ* gene. |
| pBC0989’*Z* | The promoter region of BC0989 gene was amplified using primers BC0989F/ BC0989R with 407 chromosomal DNA as template and inserted between the *Pst*I and *Xba*I sites of pHT304.18Ω*lacZ*. This results in the creation of a transcriptional fusion between BC0989 promoter and *lacZ* gene. |
| pBC2141’*Z* | The promoter region of BC2141 gene was amplified using primers BC2141F/ BC2141R with 407 chromosomal DNA as template and inserted between the *Pst*I and *Xba*I sites of pHT304.18Ω*lacZ*. This results in the creation of a transcriptional fusion between BC2141 promoter and *lacZ* gene. |
| pBC2167’*Z* | The promoter region of BC2167 gene was amplified using primers BC2167F/ BC2167R with 407 chromosomal DNA as template and inserted between the *Pst*I and *Xba*I sites of pHT304.18Ω*lacZ*. This results in the creation of a transcriptional fusion between BC2167 promoter and *lacZ* gene. |
| pBC2450’*Z* | The promoter region of BC2450 gene was amplified using primers BC2450F/ BC2450R with 407 chromosomal DNA as template and inserted between the *Pst*I and *Xba*I sites of pHT304.18Ω*lacZ*. This results in the creation of a transcriptional fusion between BC2450 promoter and *lacZ* gene. |
| pBC2682’Z | The promoter region of BC2682 gene was amplified using primers BC2682F/ BC2682R with 407 chromosomal DNA as template and inserted between the *Pst*I and *Xba*I sites of pHT304.18Ω*lacZ*. This results in the creation of a transcriptional fusion between BC2682 promoter and *lacZ* gene. |
| pBC2775’Z | The promoter region of BC2775 gene was amplified using primers BC2775F/ BC2775R with 407 chromosomal DNA as template and inserted between the *Pst*I and *Xba*I sites of pHT304.18Ω*lacZ*. This results in the creation of a transcriptional fusion between BC2775 promoter and *lacZ* gene. |
| pBC2984’Z | The promoter region of BC2984 gene was amplified using primers BC2984F/ BC2984R with 407 chromosomal DNA as template and inserted between the *Hind*III and *Bam*HI sites of pHT304.18Ω*lacZ*. This results in the creation of a transcriptional fusion between BC2984 promoter and *lacZ* gene. |
| pBC5036’Z | The promoter region of BC5036 gene was amplified using primers BC5036F/ BC5036R with 407 chromosomal DNA as template and inserted between the *Pst*I and *Xba*I sites of pHT304.18Ω*lacZ*. This results in the creation of a transcriptional fusion between BC5036 promoter and *lacZ* gene. |

Table S5
