# Supplementary figures and images for: Correction: Necrotrophism Is a Quorum-Sensing-Regulated Lifestyle in Bacillus thuringiensis
Source: PLoS Pathog. 2016 Nov 29;12(11):e1006049. doi: 10.1371/journal.ppat.1006049 (PMC5127582; doi:10.1371/journal.ppat.1006049)

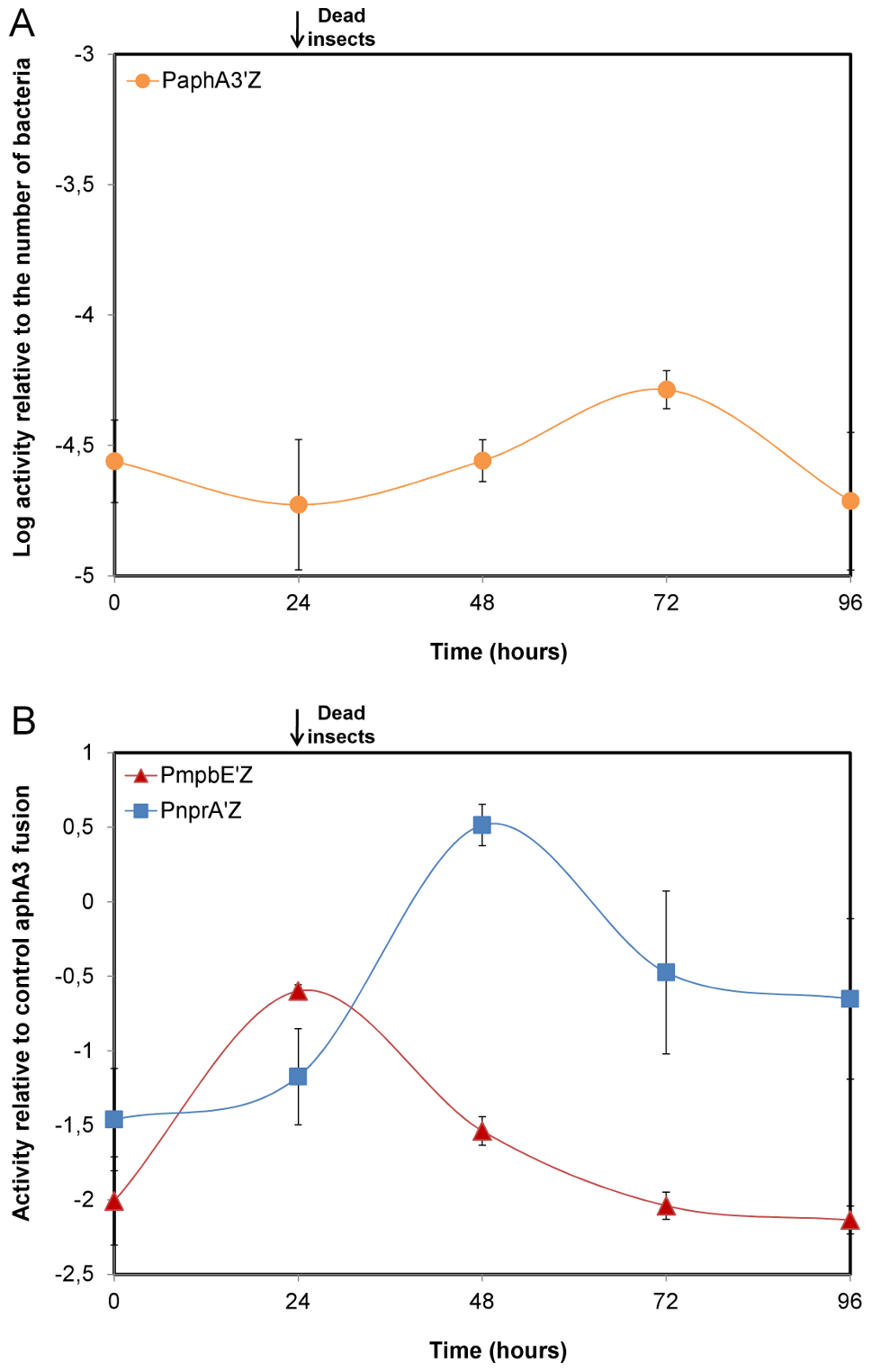

Supplement: S3 File — (ZIP) [file ppat.1006049.s003.zip › Figure 1.TIF]

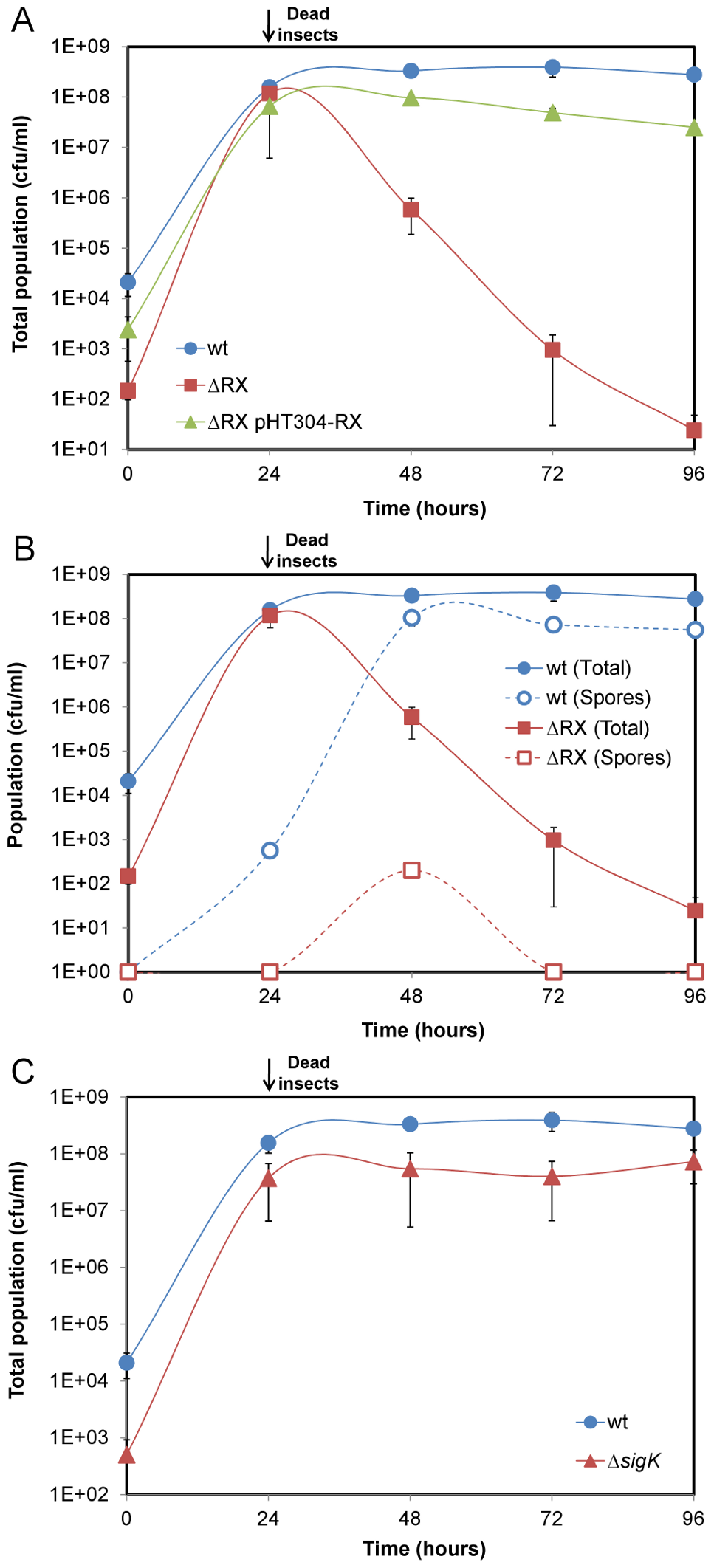

Supplement: S3 File — (ZIP) [file ppat.1006049.s003.zip › Figure 2.TIF]

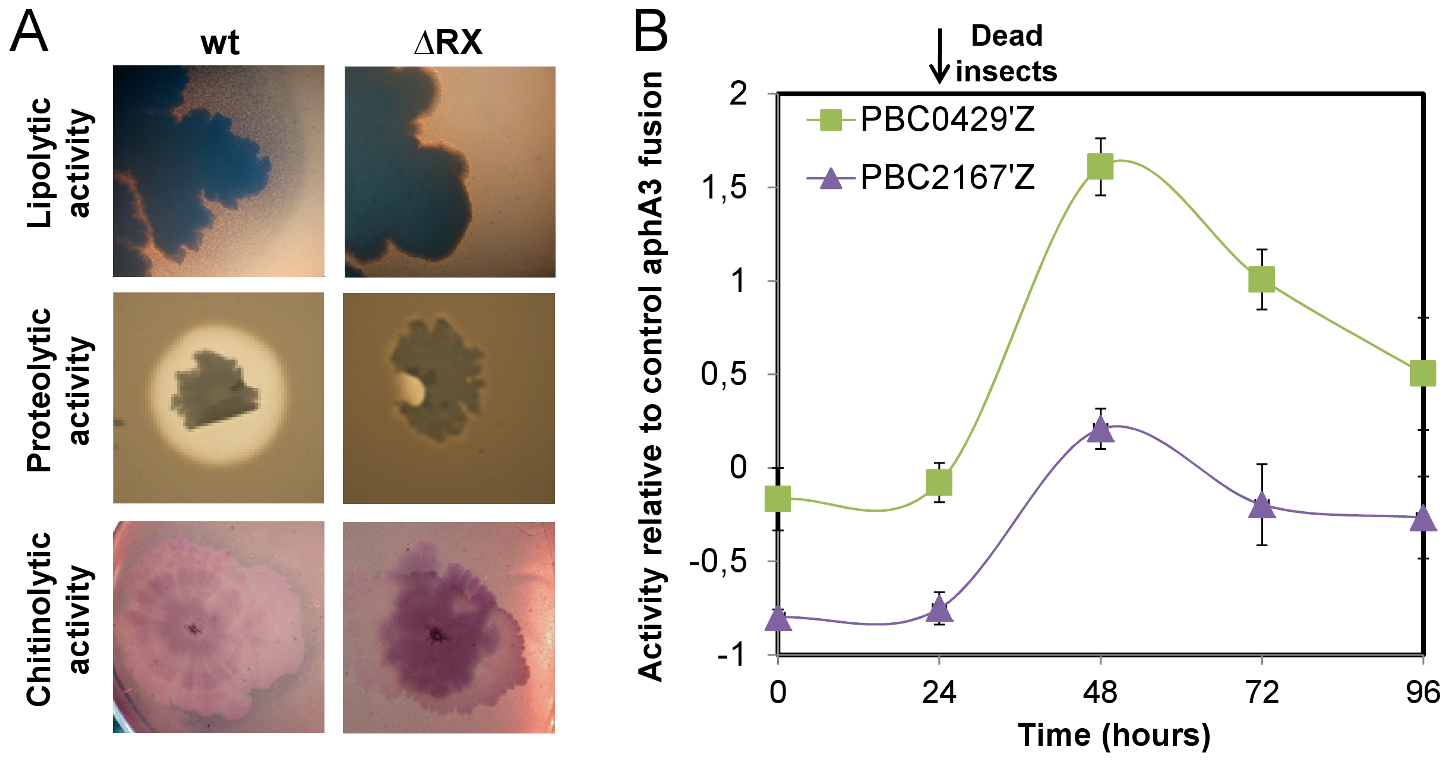

Supplement: S3 File — (ZIP) [file ppat.1006049.s003.zip › Figure 3.TIF]

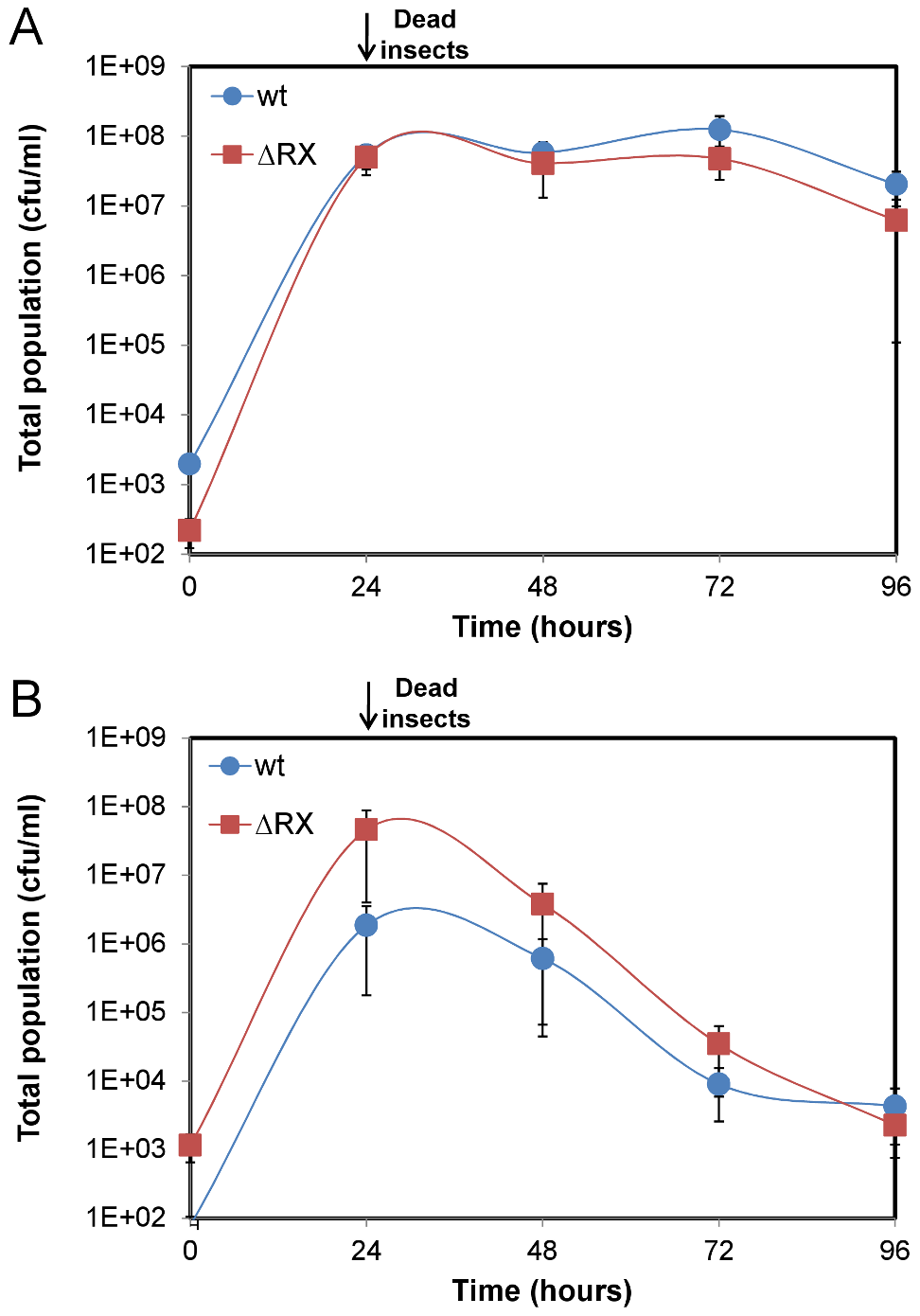

Supplement: S3 File — (ZIP) [file ppat.1006049.s003.zip › Figure 4.TIF]

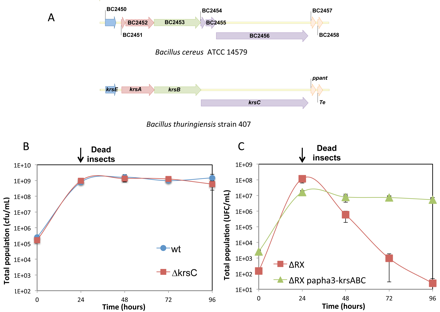

Supplement: S3 File — (ZIP) [file ppat.1006049.s003.zip › Figure 5.tiff]

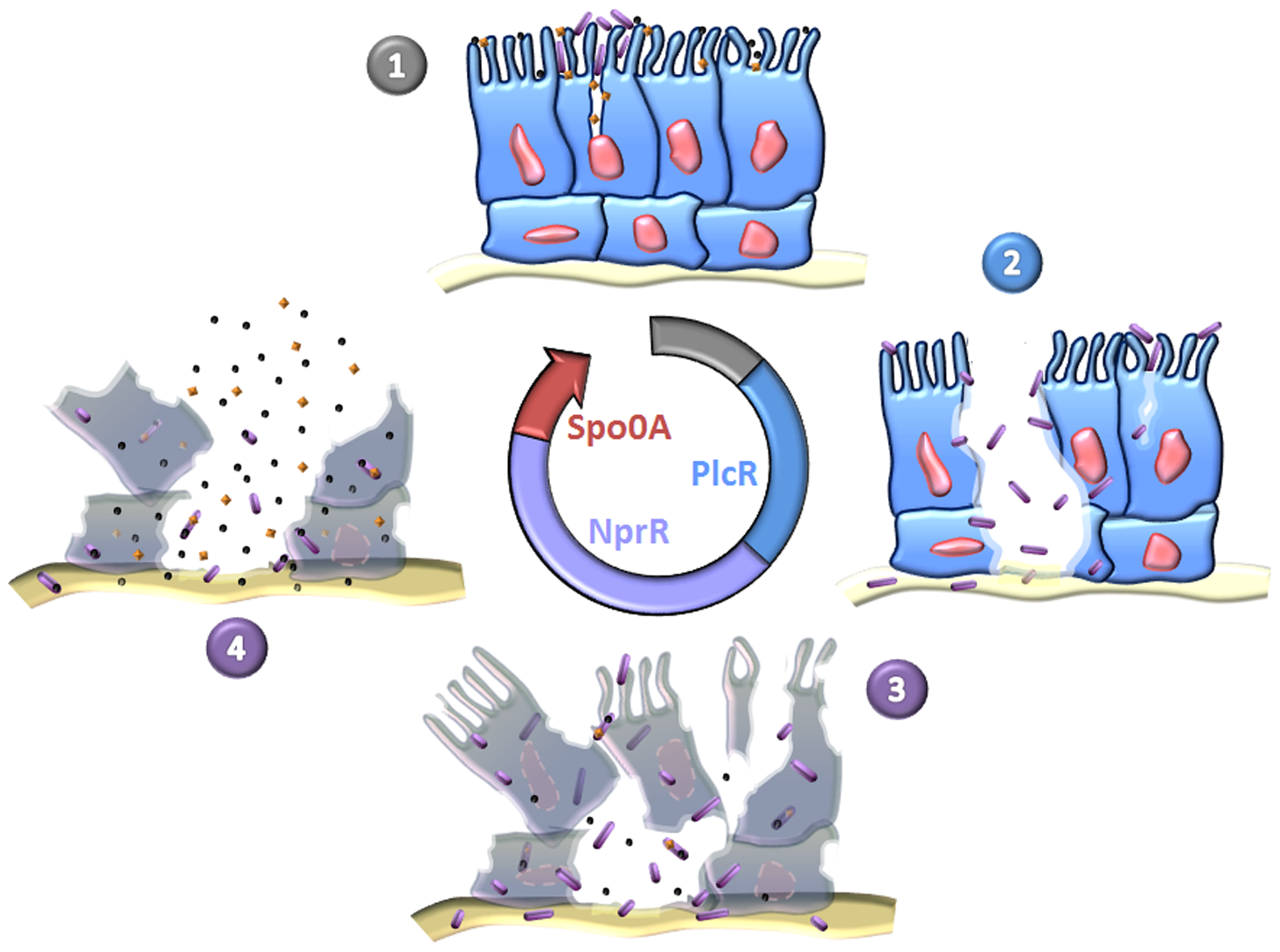

Supplement: S3 File — (ZIP) [file ppat.1006049.s003.zip › Figure 6.TIF]
